# Supplementary material for: Switchable DNA‐Based Peroxidases Controlled by a Chaotropic Ion
Source: Chembiochem. 2022 Mar 23;23(9):e202200090. doi: 10.1002/cbic.202200090 (PMC9310614; doi:10.1002/cbic.202200090)

ChemBioChem

Supporting Information

Switchable DNA-Based Peroxidases Controlled by a Chaotropic Ion**

Tanner G. Hoog[+], Matthew R. Pawlak[+], Lauren M. Aufdembrink, Benjamin R. Bachan, Matthew B. Galles, Nicholas B. Bense, Katarzyna P. Adamala, and Aaron E. Engelhart*

Table of Contents

Supplementary Figure 1. **Fluorescein-G4-Quencher** and **Duplex-Dark** melting temperatures in a series of 2 M Hofmeister salt solutions.

Supplementary Figure 2. Fluorescence melting curve (second heating trace) of **Duplex-Dark** in 0.1 M $LiClO_4$.

Supplementary Figure 3. Fluorescence melting curve (second heating trace) of **Duplex-Dark** in 0.5 M $LiClO_4$.

Supplementary Figure 4. Fluorescence melting curve (second heating trace) of **Duplex-Dark** in 1 M $LiClO_4$.

Supplementary Figure 5. Fluorescence melting curve (second heating trace) of **Duplex-Dark** in 2 M $LiClO_4$.

Supplementary Figure 6. Fluorescence melting curve (second heating trace) of **Duplex-Dark** in 3 M $LiClO_4$.

Supplementary Figure 7. Fluorescence melting curve (second heating trace) of **Duplex-Dark** in 4 M $LiClO_4$.

Supplementary Figure 8. Fluorescence melting curve (second heating trace) of **Fluorescein-G4-Quencher** in 0.1 M $NaClO_4$.

Supplementary Figure 9. Fluorescence melting curve (second heating trace) of **Fluorescein-G4-Quencher** in 0.5 M $NaClO_4$.

Supplementary Figure 10. Fluorescence melting curve (second heating trace) of **Fluorescein-G4-Quencher** in 1 M $NaClO_4$.

Supplementary Figure 11. Fluorescence melting curve (second heating trace) of **Fluorescein-G4-Quencher** in 2 M $NaClO_4$.

Supplementary Figure 12. Fluorescence melting curve (second heating trace) of **Fluorescein-G4-Quencher** in 3 M $NaClO_4$.

Supplementary Figure 13. Fluorescence melting curve (second heating trace) of **Fluorescein-G4-Quencher** in 4 M $NaClO_4$.

Supplementary Figure 14. Fluorescence melting curve (second heating trace) of **Fluorescein-G4-Quencher** in 5 M $NaClO_4$.

Supplementary Figure 15. Fluorescence melting curve (second heating trace) of **Fluorescein-G4-Quencher** in 6 M $NaClO_4$.

Supplementary Figure 16. Fluorescence melting curve (second heating trace) of **Fluorescein-G4-Quencher** in 7 M $NaClO_4$.

Supplementary Figure 17. Fluorescence melting curve (second heating trace) of **Fluorescein-G4-Quencher** in 8 M $NaClO_4$.

Supplementary Figure 18. Fluorescence melting curve (second heating trace) of **Fluorescein-G4-Quencher** in Saturated $NaClO_4$.

Supplementary Figure 19. Fluorescence melting curve (second heating trace) of **G4-Dark** in 0.1 M $NaClO_4$.

Supplementary Figure 20. Fluorescence melting curve (second heating trace) of **G4-Dark** in 0.5 M $NaClO_4$.

Supplementary Figure 21. Fluorescence melting curve (second heating trace) of **G4-Dark** in 1 M $NaClO_4$.

Supplementary Figure 22. Fluorescence melting curve (second heating trace) of **G4-Dark** in 2 M $NaClO_4$.

Supplementary Figure 23. Fluorescence melting curve (second heating trace) of **G4-Dark** in 3 M $NaClO_4$.

Supplementary Figure 24. Fluorescence melting curve (second heating trace) of **G4-Dark** in 4 M $NaClO_4$.

Supplementary Figure 25. Fluorescence melting curve (second heating trace) of **G4-Dark** in 5 M $NaClO_4$.

Supplementary Figure 26. Fluorescence melting curve (second heating trace) of **G4-Dark** in 6 M $NaClO_4$.

Supplementary Figure 27. Fluorescence melting curve (second heating trace) of **G4-Dark** in 7 M $NaClO_4$.

Supplementary Figure 28. Fluorescence melting curve (second heating trace) of **G4-Dark** in 8 M $NaClO_4$.

Supplementary Figure 29. Fluorescence melting curve (second heating trace) of **G4-Dark** in Saturated $NaClO_4$.

Supplementary Figure 30. Engineering drawing of 3D printed tube holder/camera mount component of imaging jig. Dimensions in mm.

Supplementary Figure 31. Engineering drawing of 3D printed led holder component of imaging jig. Dimensions in mm.

Supplementary Figure 32. CAD Mockup of Fluorescence Imaging Jig

Supplementary Figure 33. CAD Mockup of Fluorescence Imaging Jig (Alternate view)

Supplementary Figure 34. Imaging jig with tubes.

Supplementary Figure 35. Imaging jig (top view).

Supplementary Figure 36. Imaging Jig (side view)

Supplementary Figure 37. Imaging Jig (front view).

Supplementary Video 1. Fluorescence Imaging shows G4-Dark and Duplex-Dark switching structure under vacuum control with real-time plotting of fluorescence intensity

Supplementary Video 2. Fluorescence Imaging shows G4-Dark and Duplex-Dark switching structure under vacuum control (With annotations and captions)

Supplementary Code 1. Python code for controlling Raspberry Pi for reaction monitoring and overlaying plots of fluorescence vs. time.

Supplementary Code 2. Python code for reanalyzing collected images.

Supplementary Code 3. Python code for generating fluorescence plot overlays for existing image datasets.

Supplementary Table 1. Melting temperature of fluorescent nucleic acid systems in low (0.8 M) and high (8 M) concentrations of sodium perchlorate with varying concentrations of oligonucleotide.

Supplementary Table 2. Thermal midpoints of **G4-SwitchR.** Measurements were obtained by UV-vis monitoring of A-$_{260}$ and A-$_{295}$.

Supplementary Figure 38. Comparative kinetic measurements of oxidation of Amplex Red by **G4Redox** and **G4-SwitchR** in the presence of varying concentrations of $NaClO_4$.

Supplementary Figure 39. UV-visible spectra of hemin interacting with **G4Redox** and **G4SwitchR** in varying sodium perchlorate concentrations.

Supplementary Figure 40. CD spectra of **G4Redox** and **G4-SwitchR** in the presence and absence of hemin.

Supplementary Figure 41. UV-vis spectra of **G4Redox** and hemin in varying sodium perchlorate concentrations .

Supplementary Figure 42. **G4-SwitchR**-hemin complexes catalyze oxidation of TMB and ABTS.

Supplementary Figure 43. A_{295}-monitored melting curve of the **G4-SwitchR** system.

Supplementary Figure 44. A_{260}-monitored melting curve of the **G4-SwitchR** system.

Supplementary Figure 1. **Fluorescein-G4-Quencher** and **Duplex-Dark** melting temperatures in a series of 2 M Hofmeister salt solutions. The anions in the salts range from kosmotropes (sodium sulfate), to Hofmeister-neutral ions (sodium chloride) to chaotropes (sodium nitrate and sodium perchlorate). Points with asterisks represent incomplete melting transitions at 95 °C.

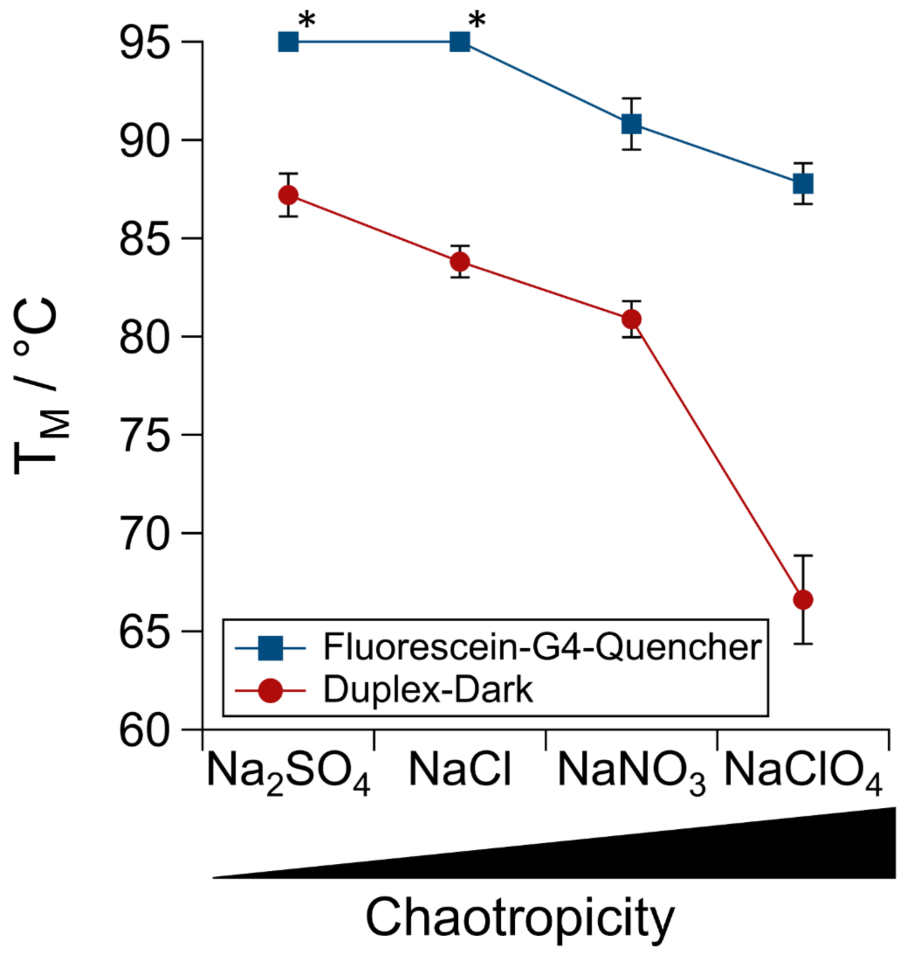

Supplementary Figure 2. Fluorescence melting curve (second heating trace) of **Duplex-Dark** in 0.1 M $LiClO_4$.

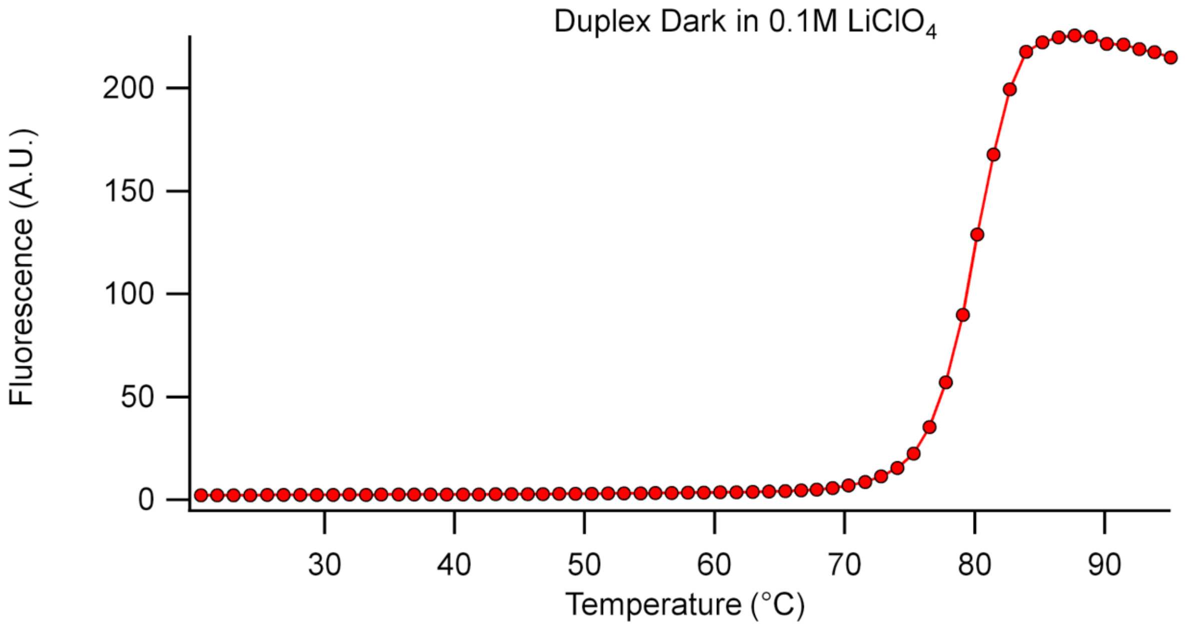

Supplementary Figure 3. Fluorescence melting curve (second heating trace) of **Duplex-Dark** in 0.5 M $LiClO_4$.

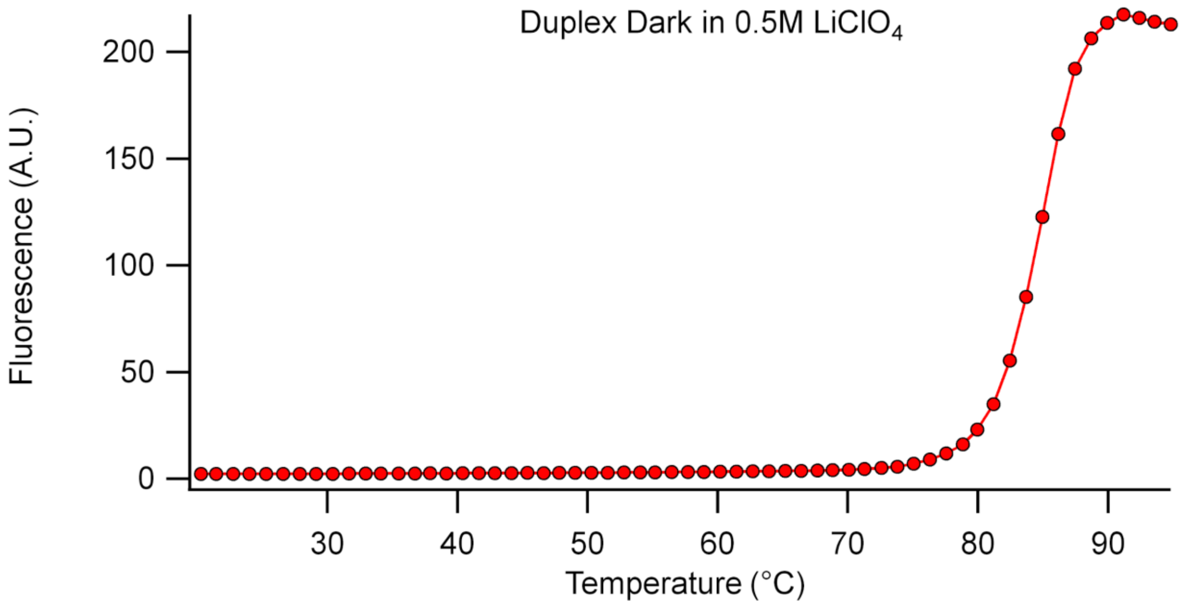

Supplementary Figure 4. Fluorescence melting curve (second heating trace) of **Duplex-Dark** in 1 M $LiClO_4$.

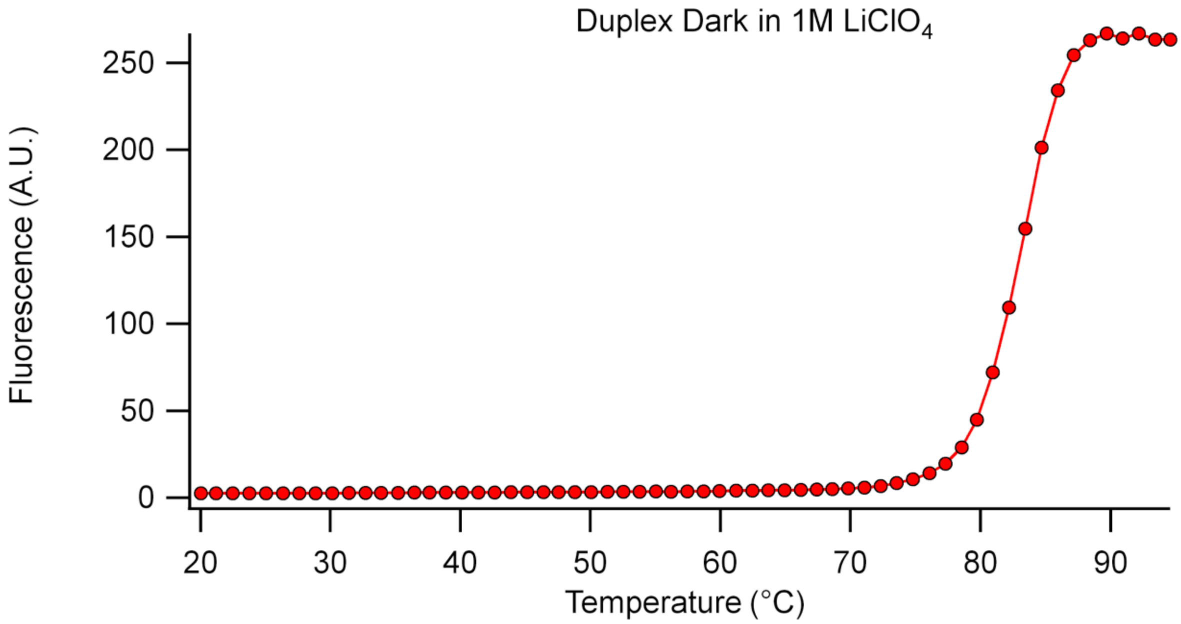

Supplementary Figure 5. Fluorescence melting curve (second heating trace) of **Duplex-Dark** in 2 M $LiClO_4$.

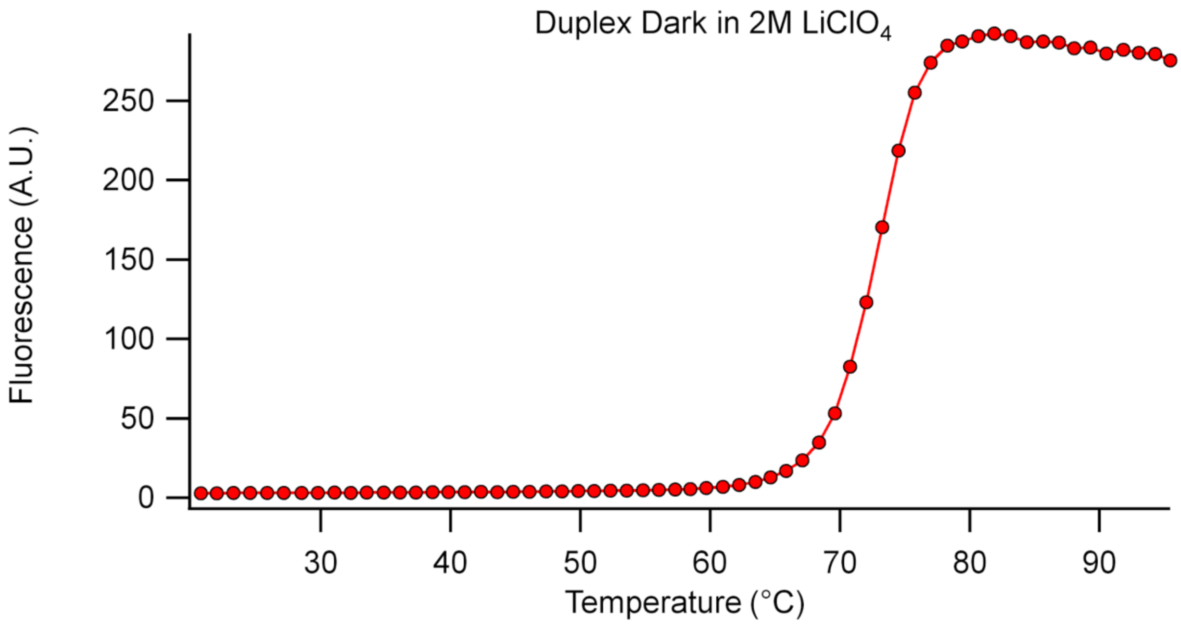

Supplementary Figure 6. Fluorescence melting curve (second heating trace) of **Duplex-Dark** in 3 M $LiClO_4$.

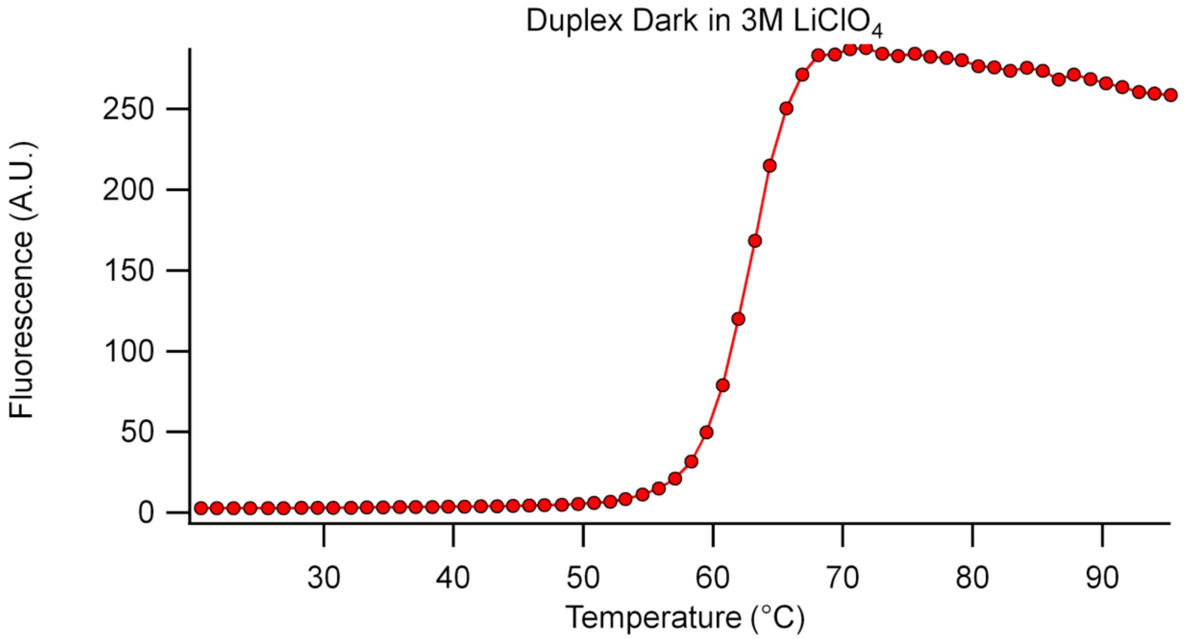

Supplementary Figure 7. Fluorescence melting curve (second heating trace) of **Duplex-Dark** in 4 M $LiClO_4$.

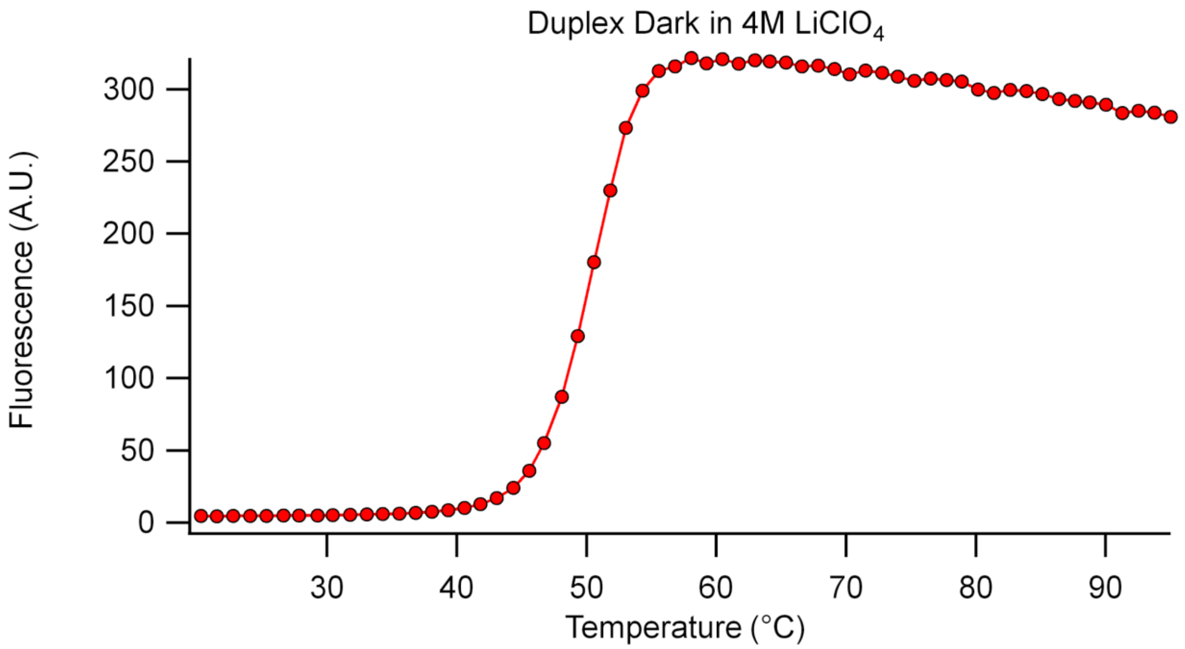

Supplementary Figure 8. Fluorescence melting curve (second heating trace) of **Fluorescein-G4-Quencher** in 0.1 M $NaClO_4$.

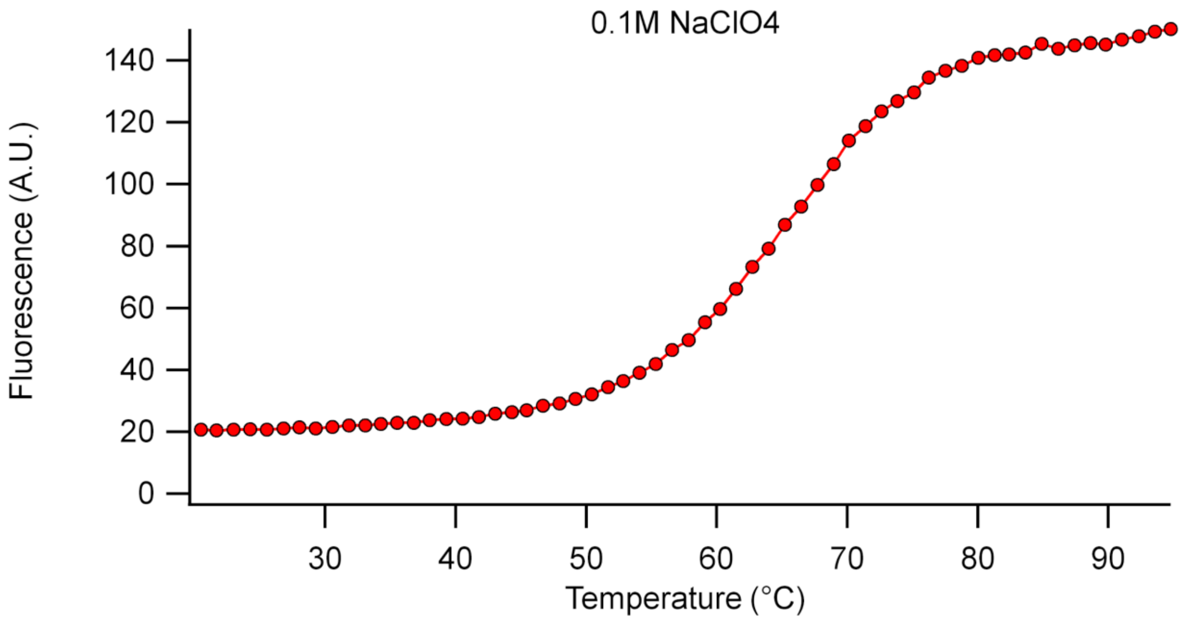

Supplementary Figure 9. Fluorescence melting curve (second heating trace) of **Fluorescein-G4-Quencher** in 0.5 M $NaClO_4$.

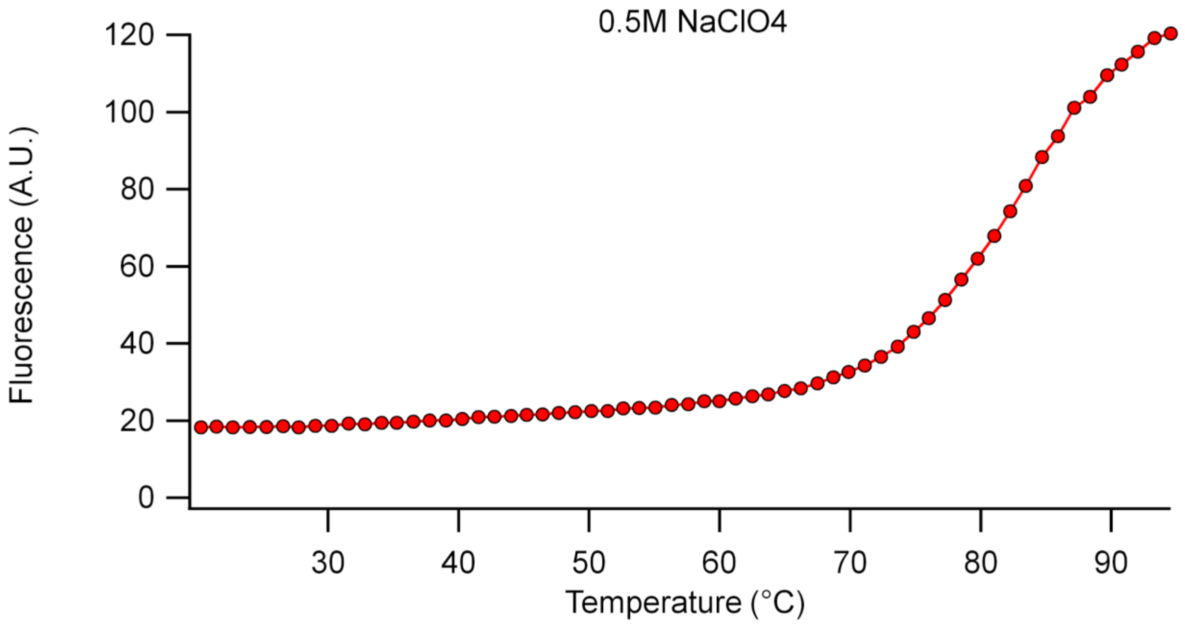

Supplementary Figure 10. Fluorescence melting curve (second heating trace) of **Fluorescein-G4-Quencher** in 1 M $NaClO_4$.

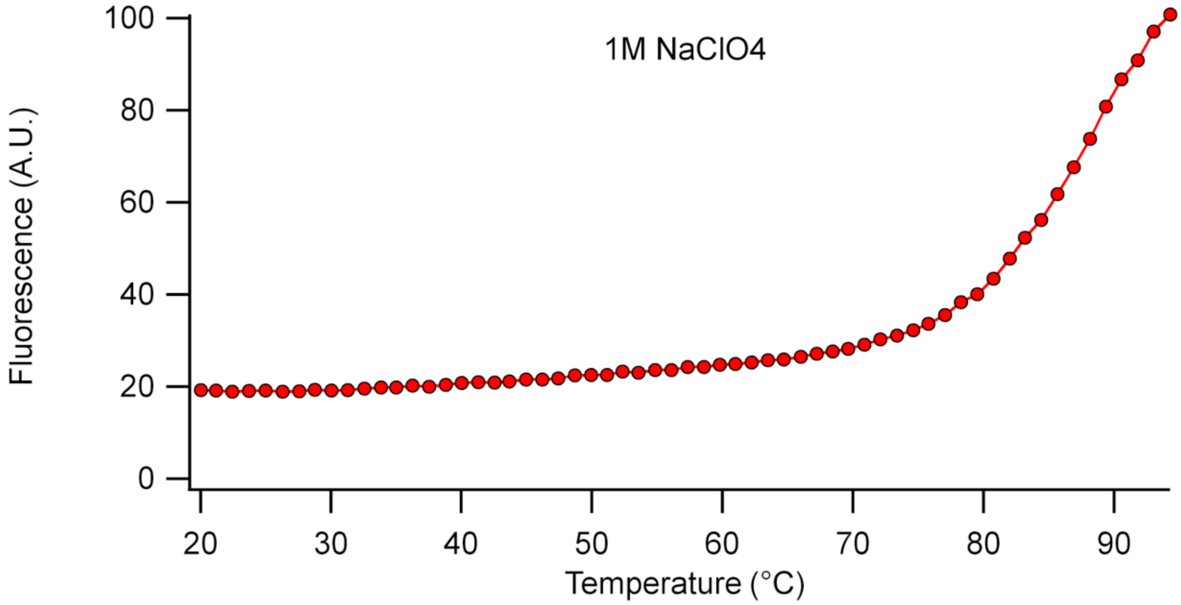

Supplementary Figure 11. Fluorescence melting curve (second heating trace) of **Fluorescein-G4-Quencher** in 2 M $NaClO_4$.

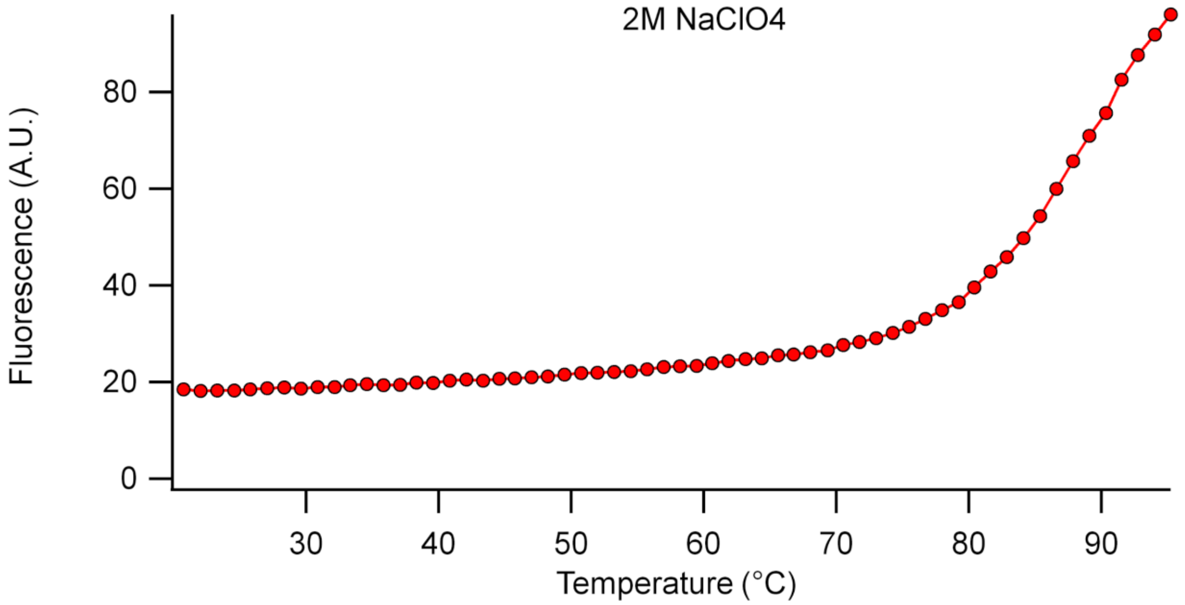

Supplementary Figure 12. Fluorescence melting curve (second heating trace) of **Fluorescein-G4-Quencher** in 3 M $NaClO_4$.

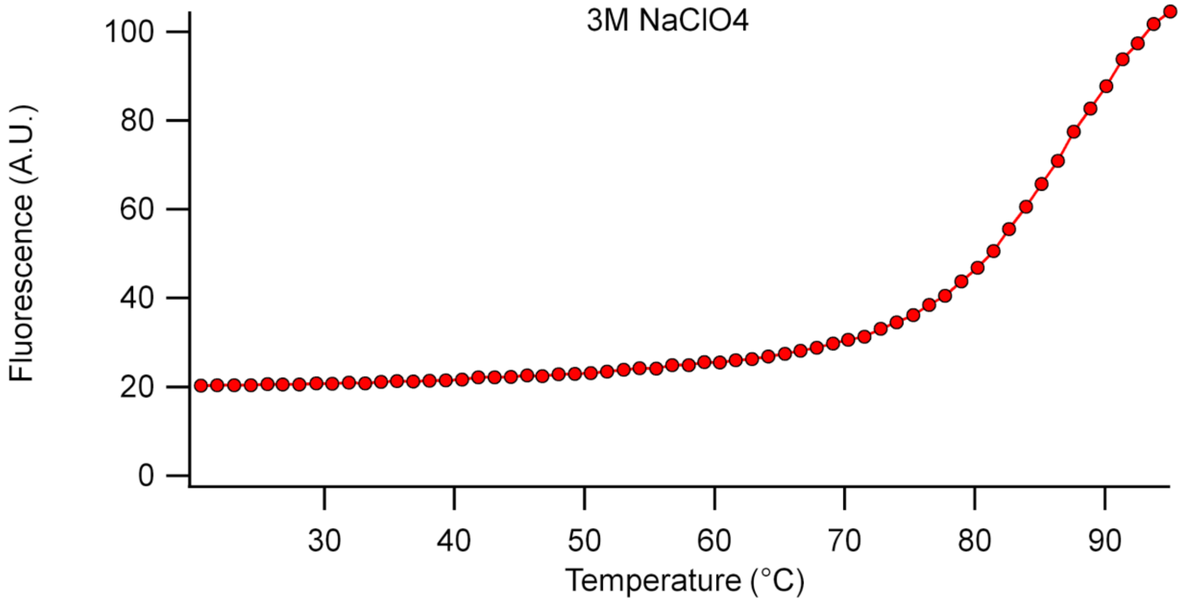

Supplementary Figure 13. Fluorescence melting curve (second heating trace) of **Fluorescein-G4-Quencher** in 4 M $NaClO_4$.

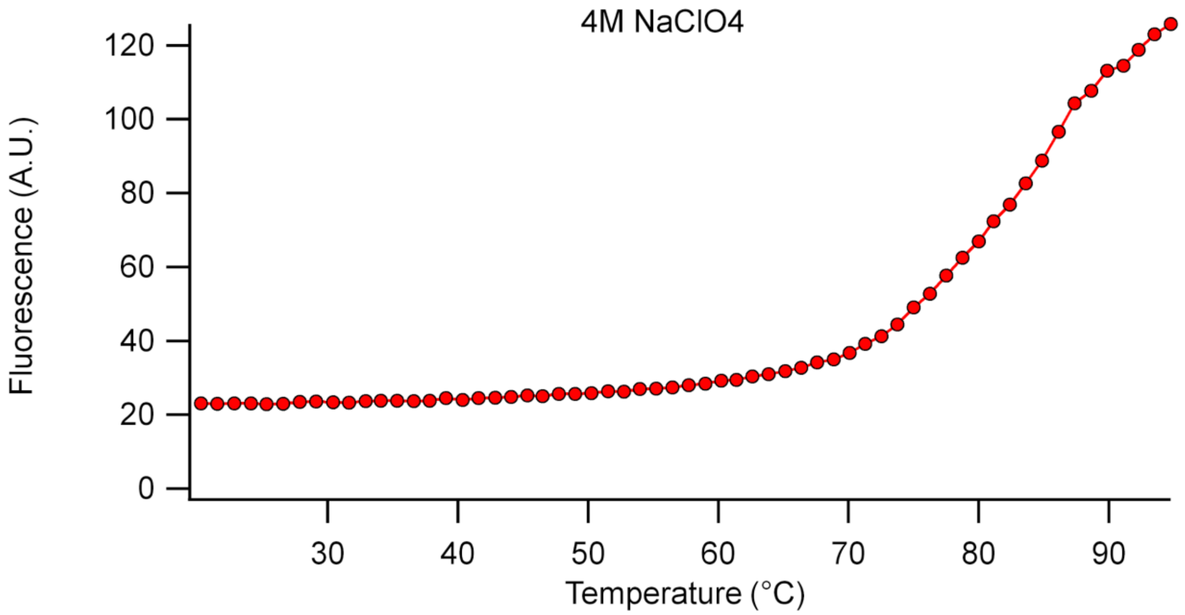

Supplementary Figure 14. Fluorescence melting curve (second heating trace) of **Fluorescein-G4-Quencher** in 5 M $NaClO_4$.

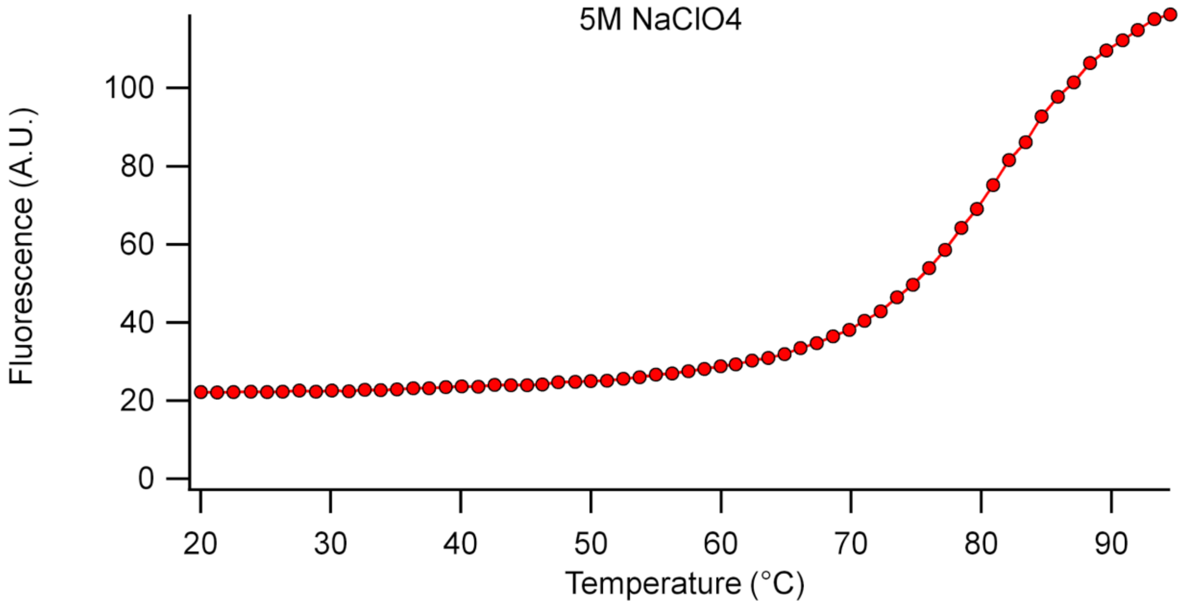

Supplementary Figure 15. Fluorescence melting curve (second heating trace) of **Fluorescein-G4-Quencher** in 6 M $NaClO_4$.

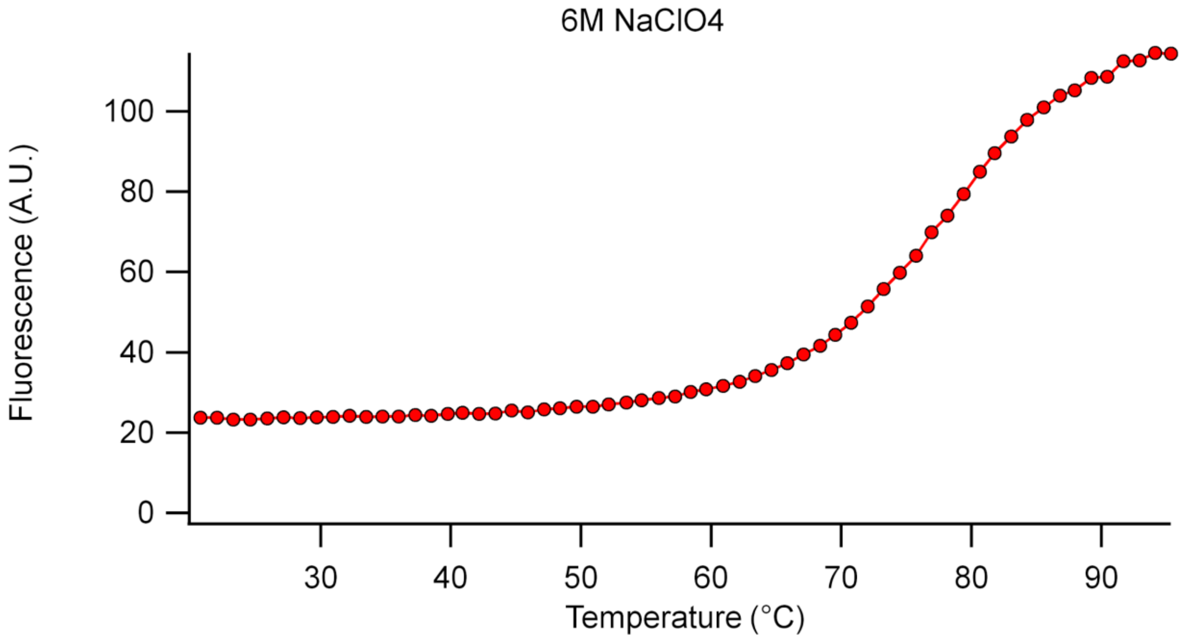

Supplementary Figure 16. Fluorescence melting curve (second heating trace) of **Fluorescein-G4-Quencher** in 7 M $NaClO_4$.

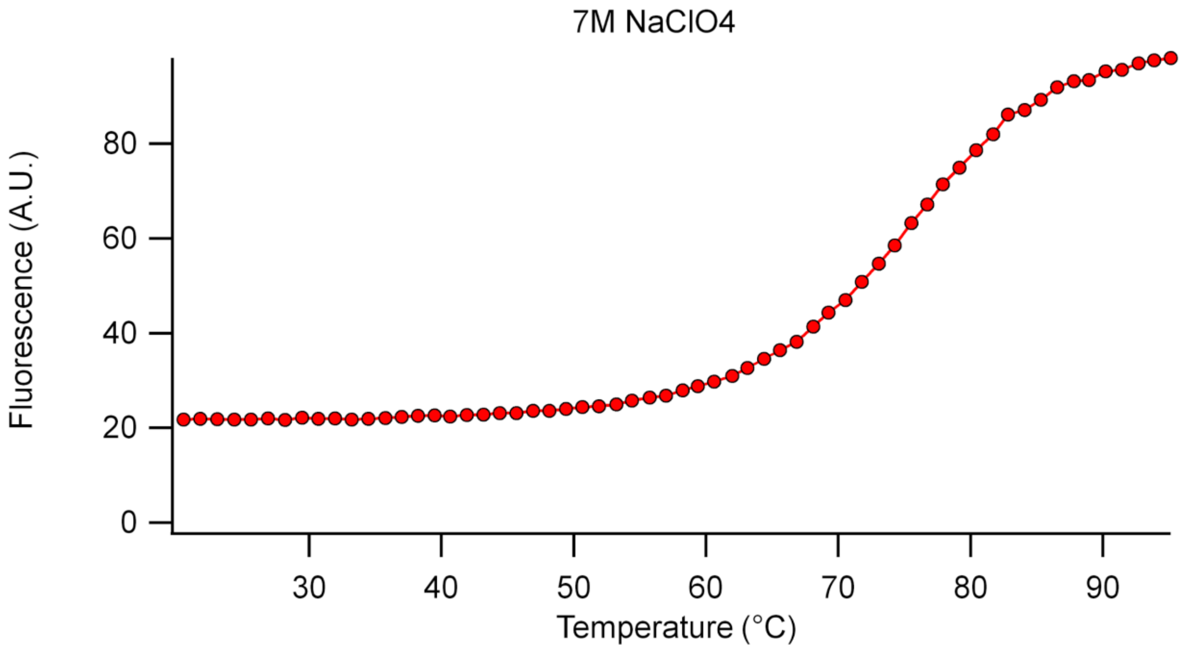

Supplementary Figure 17. Fluorescence melting curve (second heating trace) of **Fluorescein-G4-Quencher** in 8 M $NaClO_4$.

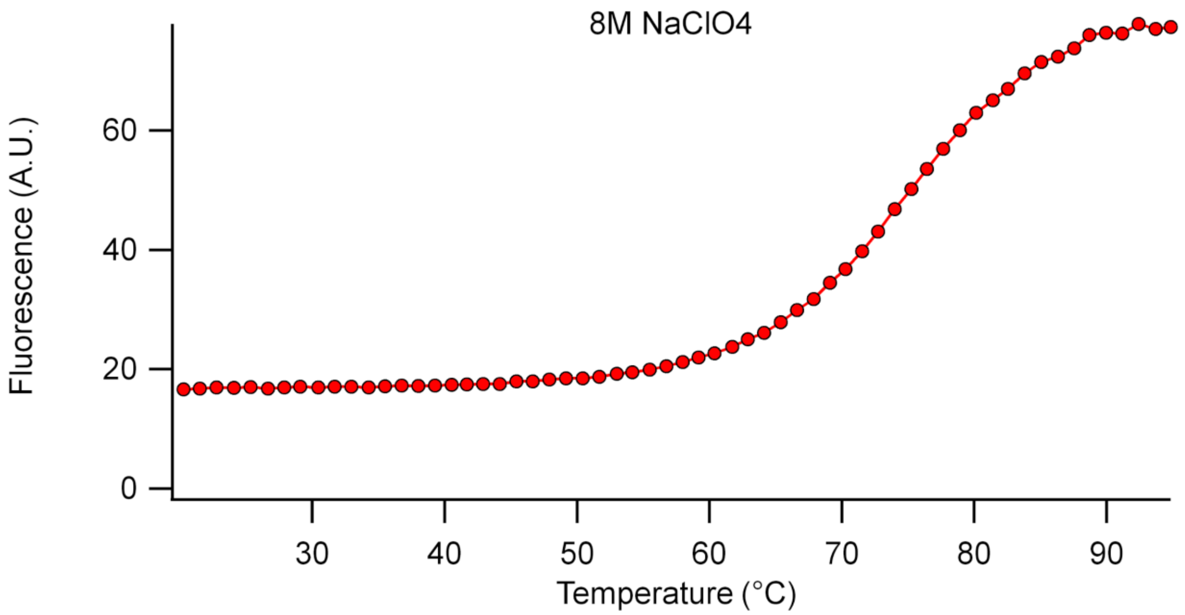

Supplementary Figure 18. Fluorescence melting curve (second heating trace) of **Fluorescein-G4-Quencher** in Saturated (ca. 9 M) $NaClO_4$.

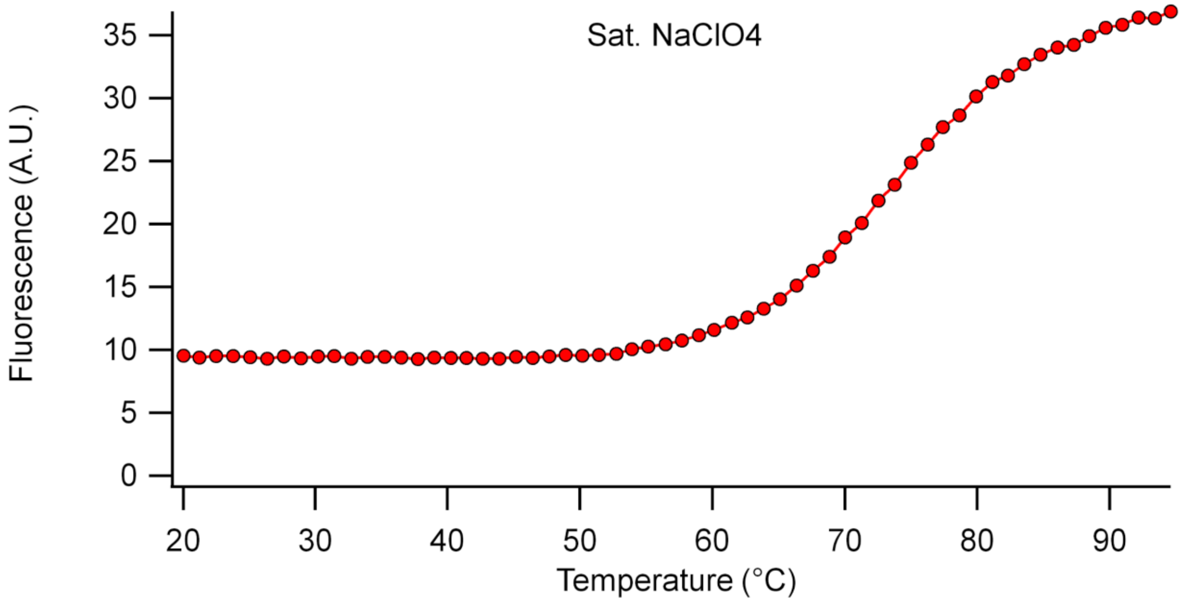

Supplementary Figure 19. Fluorescence melting curve (second heating trace) of **G4-Dark** in 0.1 M $NaClO_4$.

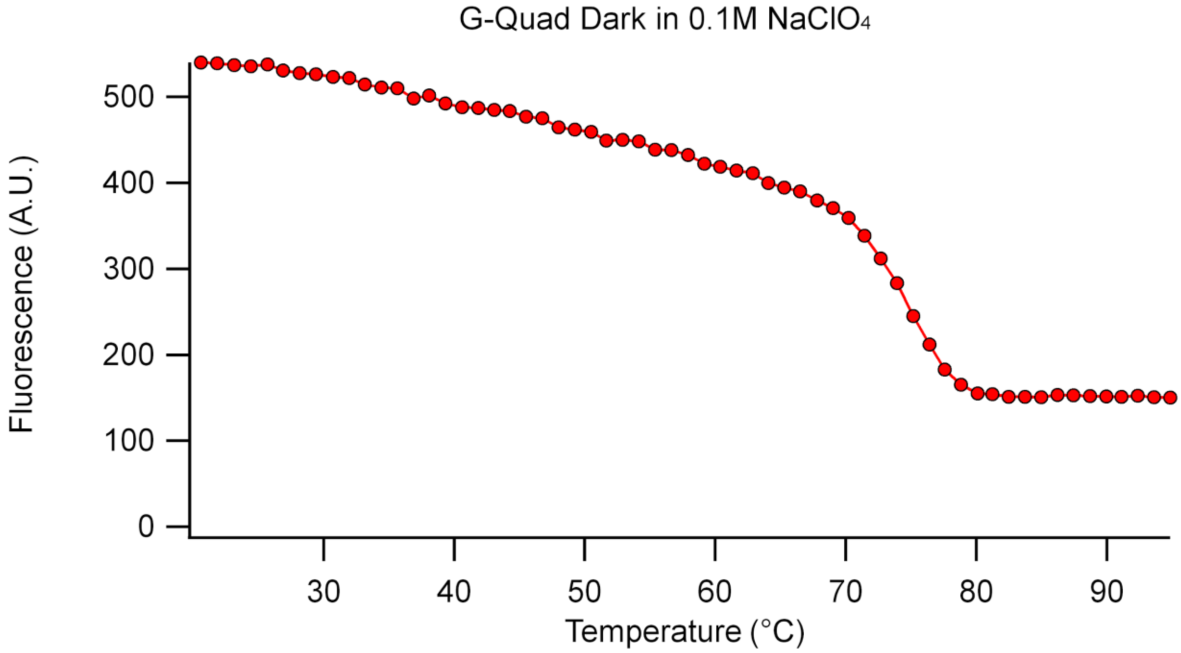

Supplementary Figure 20. Fluorescence melting curve (second heating trace) of **G4-Dark** in 0.5 M $NaClO_4$.

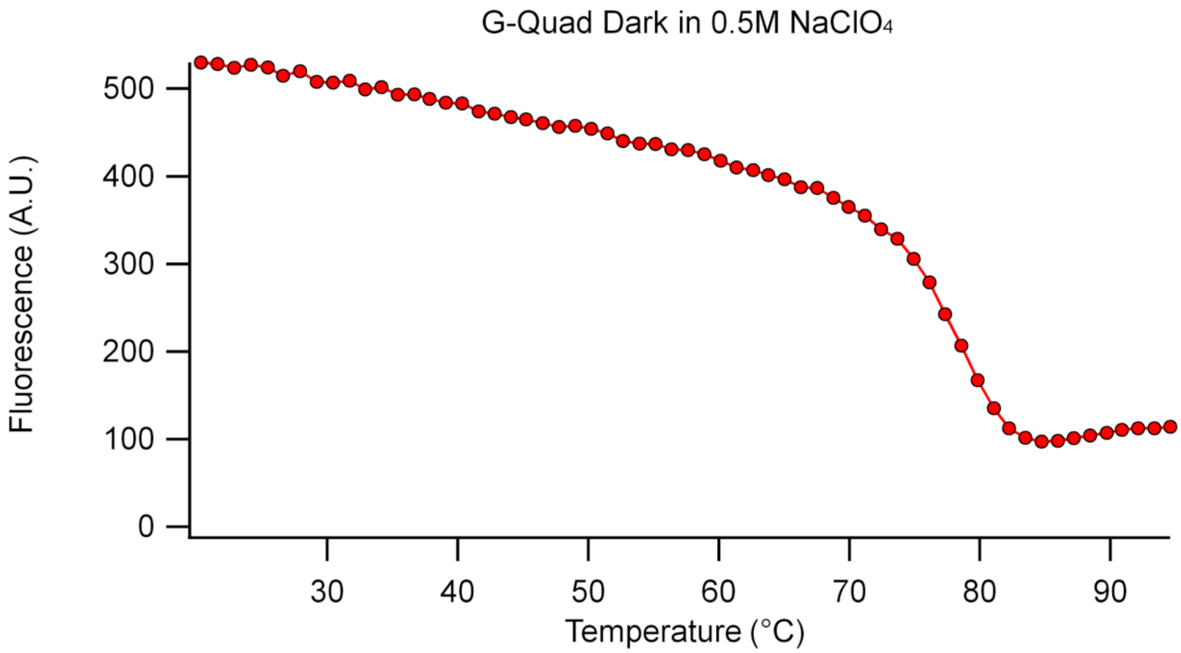

Supplementary Figure 21. Fluorescence melting curve (second heating trace) of **G4-Dark** in 1 M $NaClO_4$.

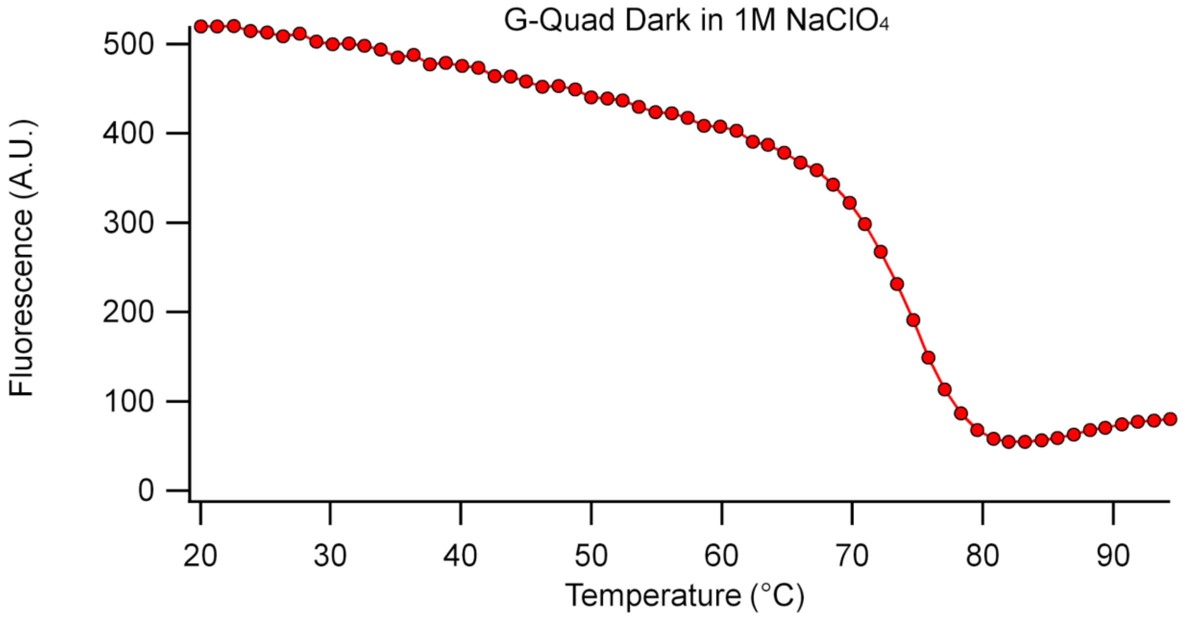

Supplementary Figure 22. Fluorescence melting curve (second heating trace) of **G4-Dark** in 2 M $NaClO_4$.

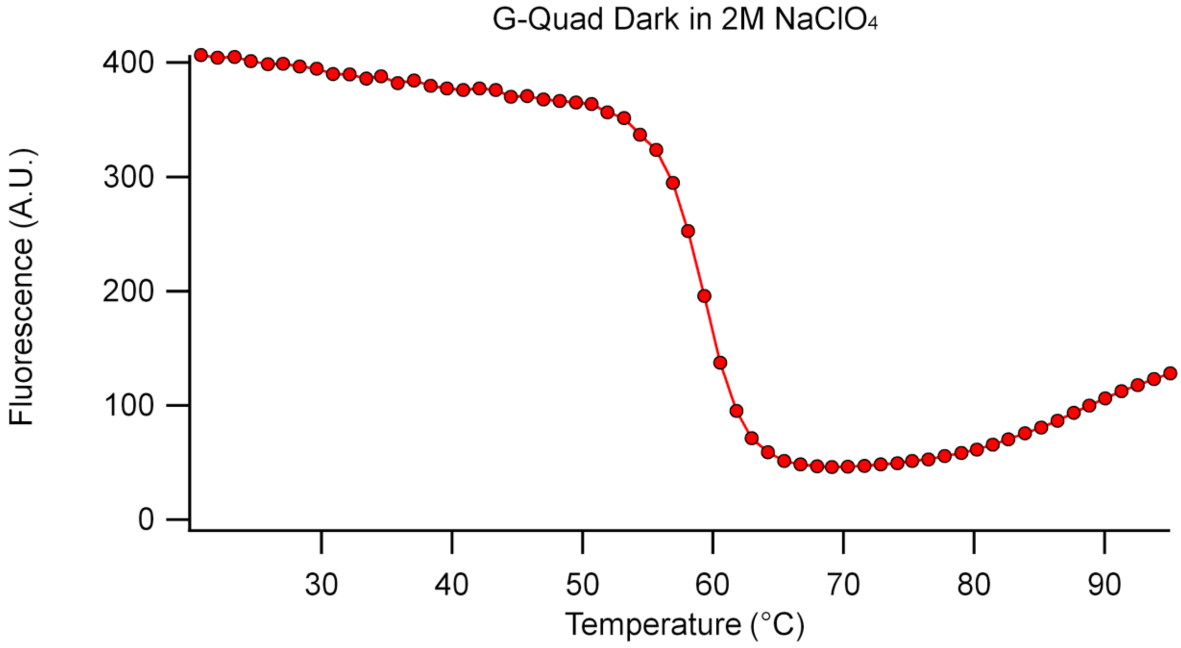

Supplementary Figure 23. Fluorescence melting curve (second heating trace) of **G4-Dark** in 3 M $NaClO_4$.

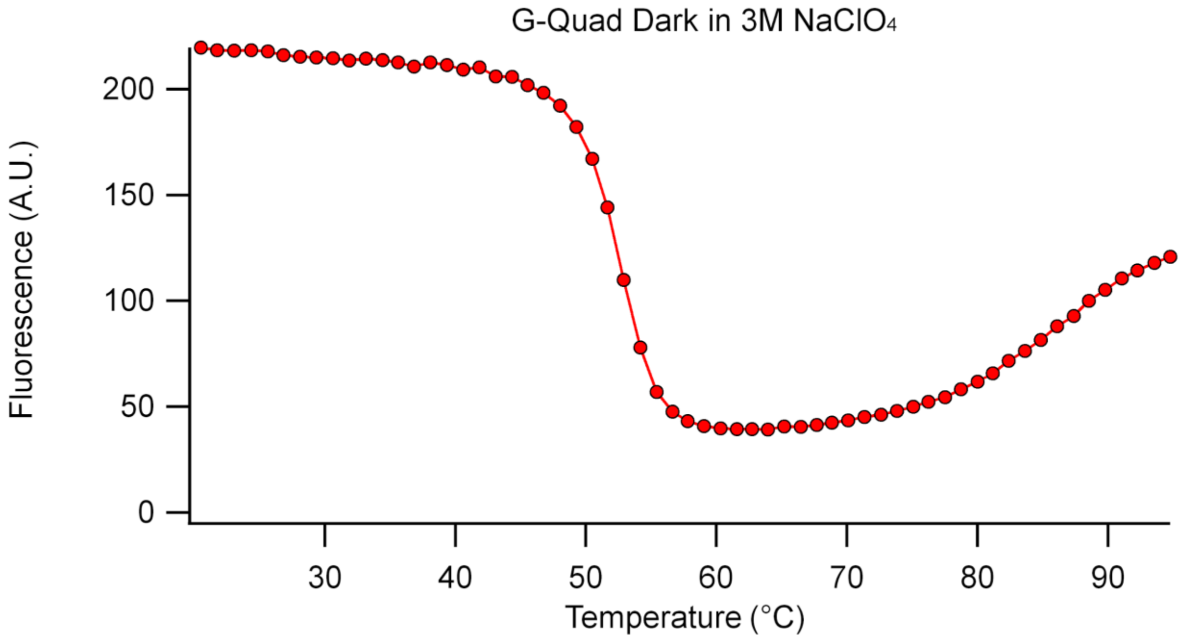

Supplementary Figure 24. Fluorescence melting curve (second heating trace) of **G4-Dark** in 4 M $NaClO_4$.

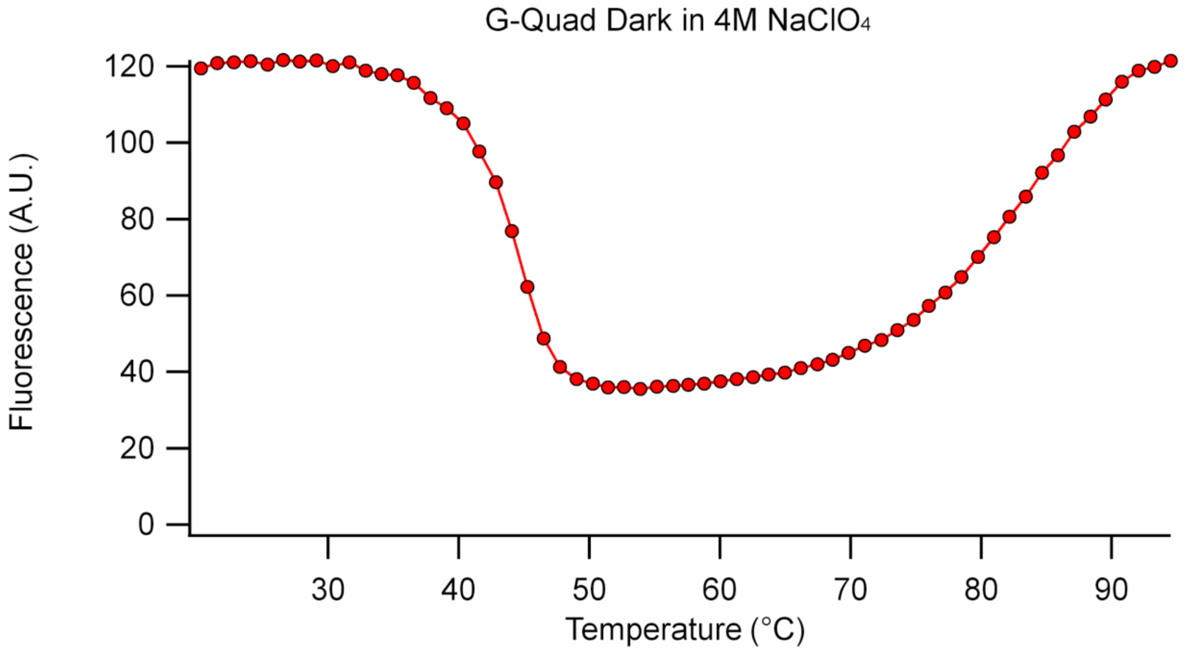

Supplementary Figure 25. Fluorescence melting curve (second heating trace) of **G4-Dark** in 5 M $NaClO_4$.

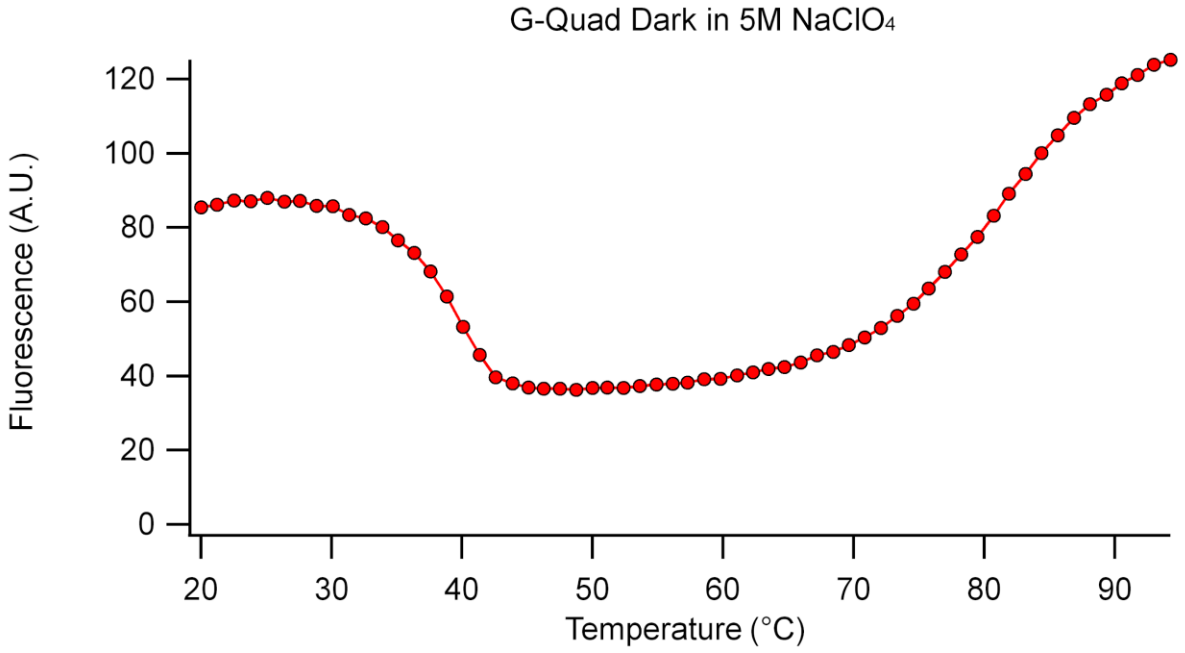

Supplementary Figure 26. Fluorescence melting curve (second heating trace) of **G4-Dark** in 6 M $NaClO_4$.

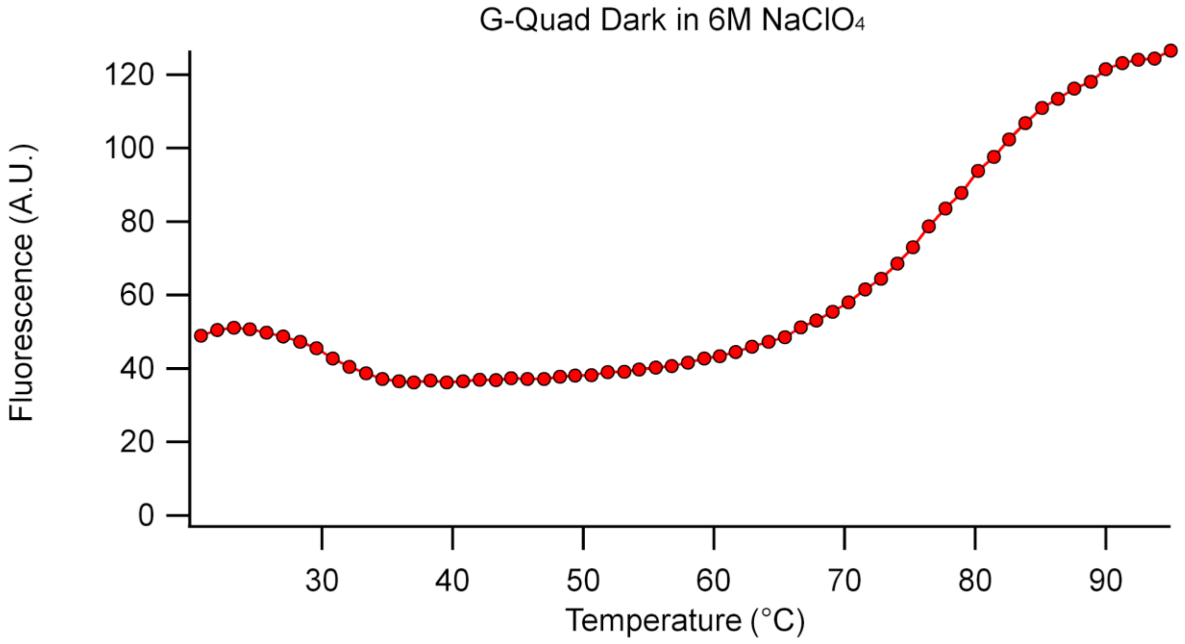

Supplementary Figure 27. Fluorescence melting curve (second heating trace) of **G4-Dark** in 7 M $NaClO_4$.

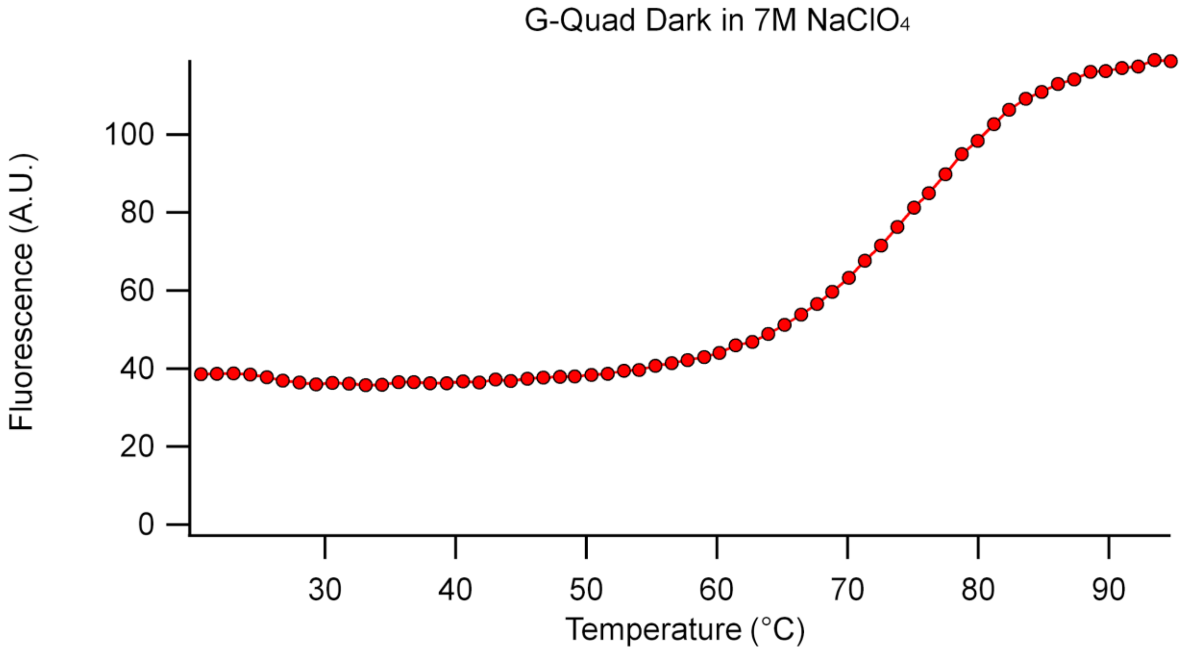

Supplementary Figure 28. Fluorescence melting curve (second heating trace) of **G4-Dark** in 8 M $NaClO_4$.

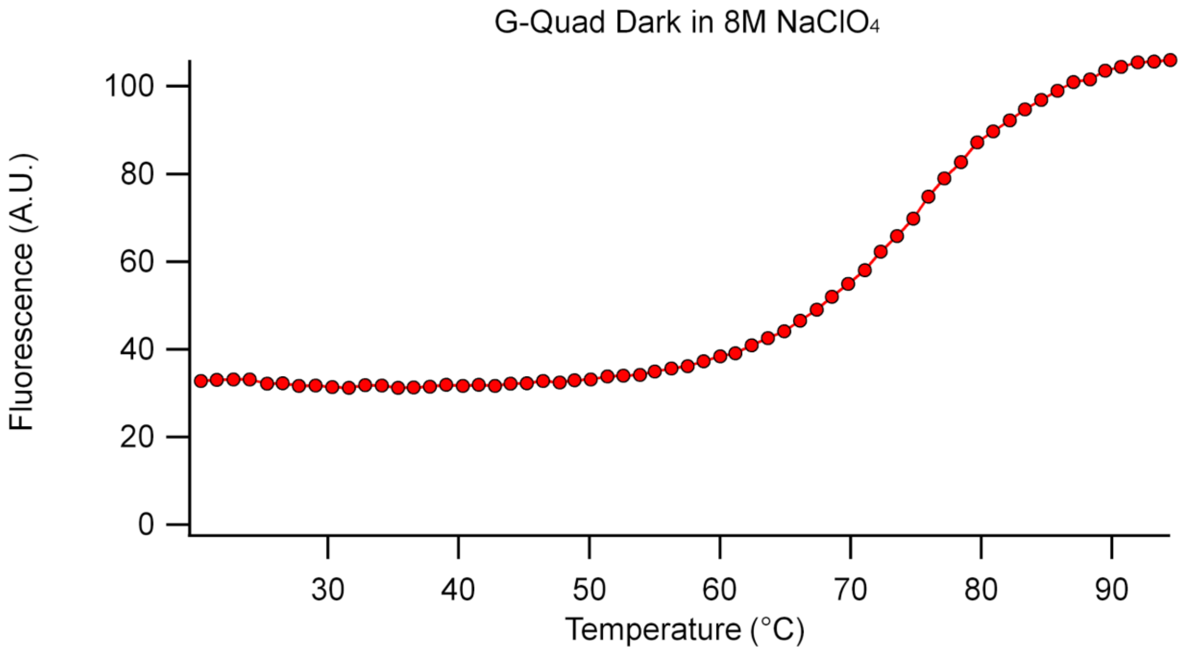

Supplementary Figure 29. Fluorescence melting curve (second heating trace) of **G4-Dark** in Saturated $NaClO_4$.

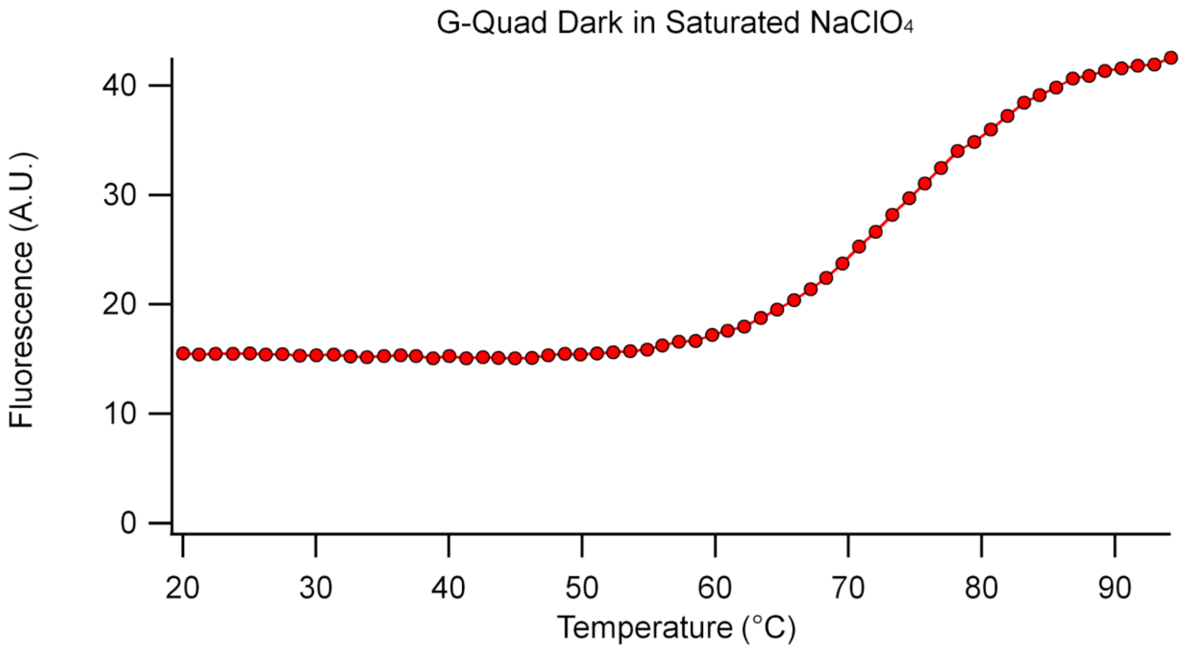

Supplementary Figure 30. Engineering drawing of 3D printed tube holder/camera mount component of imaging jig. Dimensions in mm.

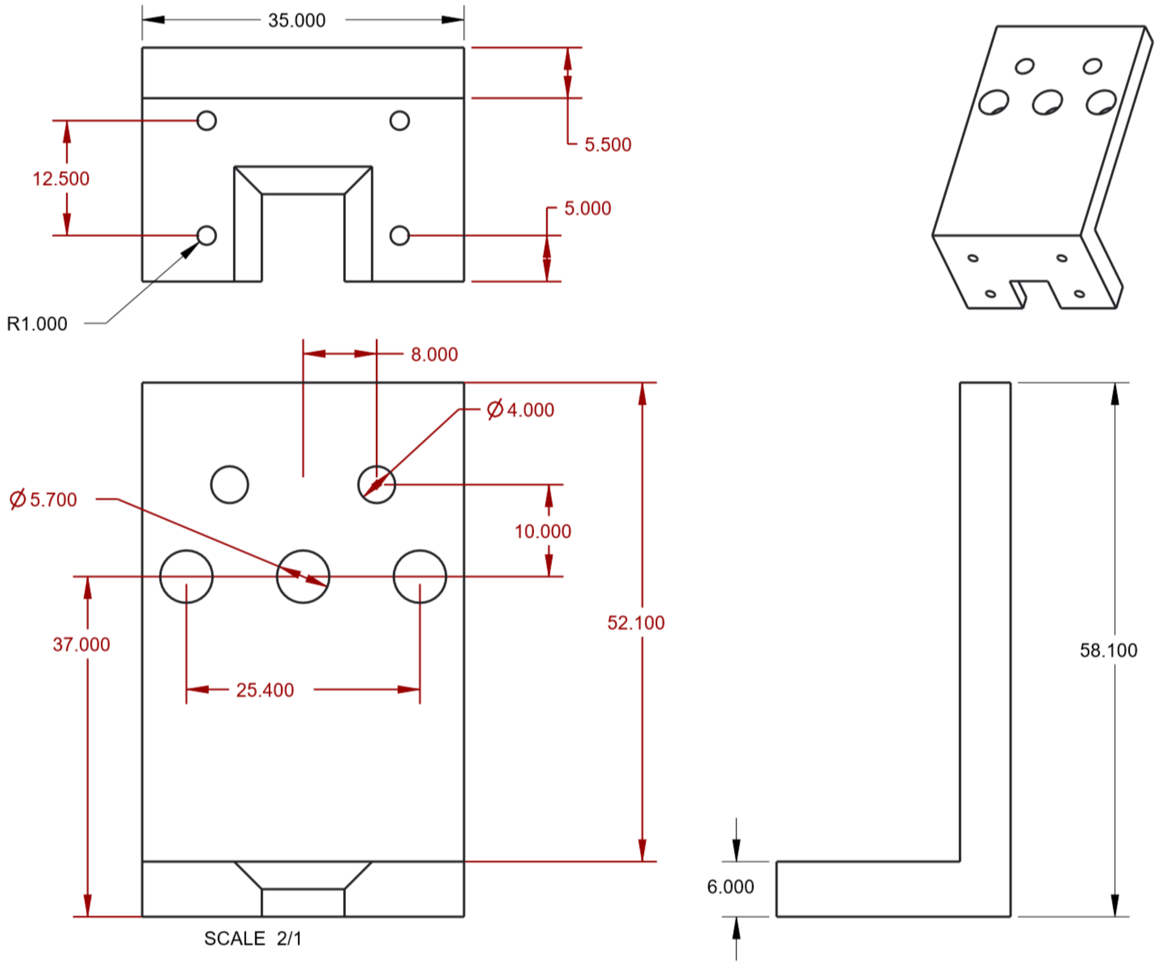

Supplementary Figure 31. Engineering drawing of 3D printed led holder component of imaging jig. Dimensions in mm.

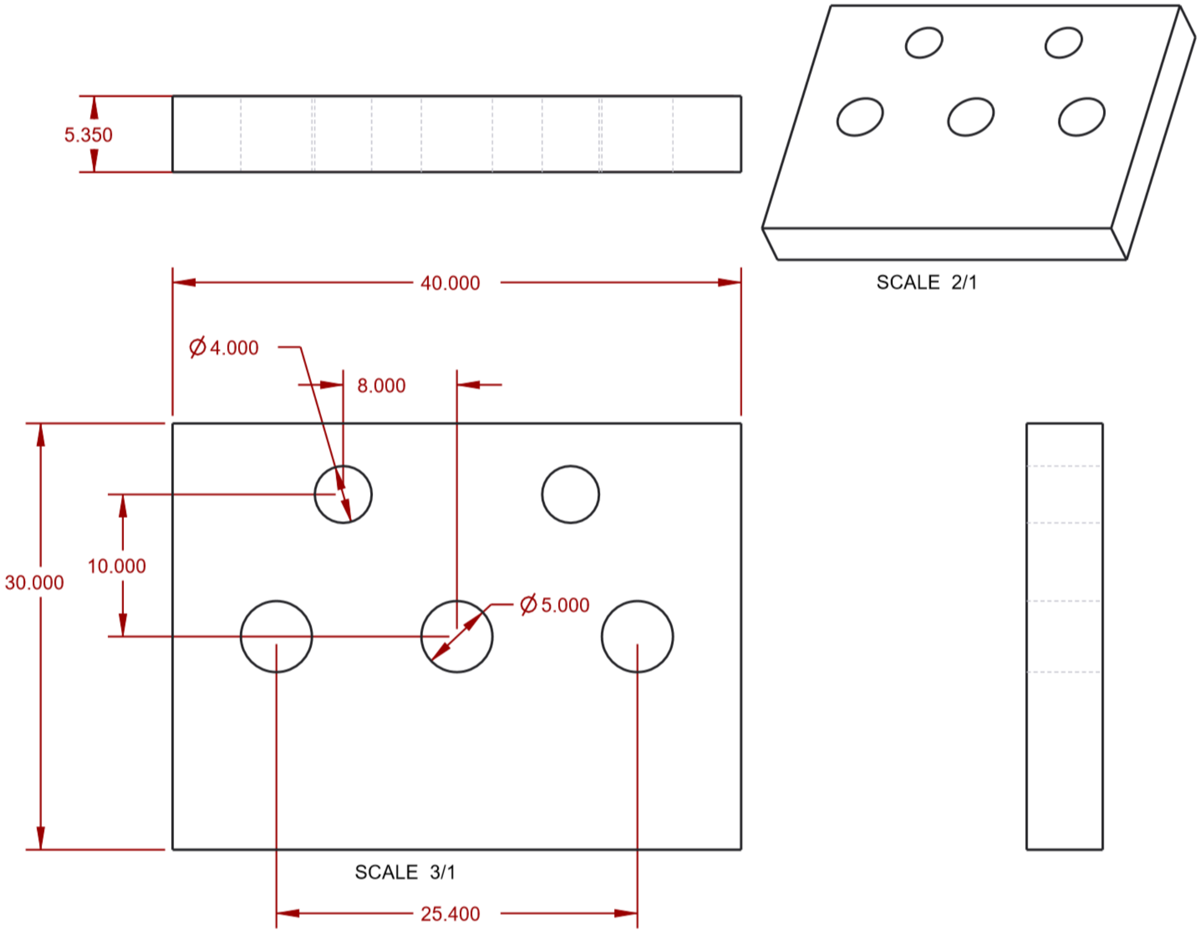

Supplementary Figure 32. CAD Mockup of Fluorescence Imaging Jig

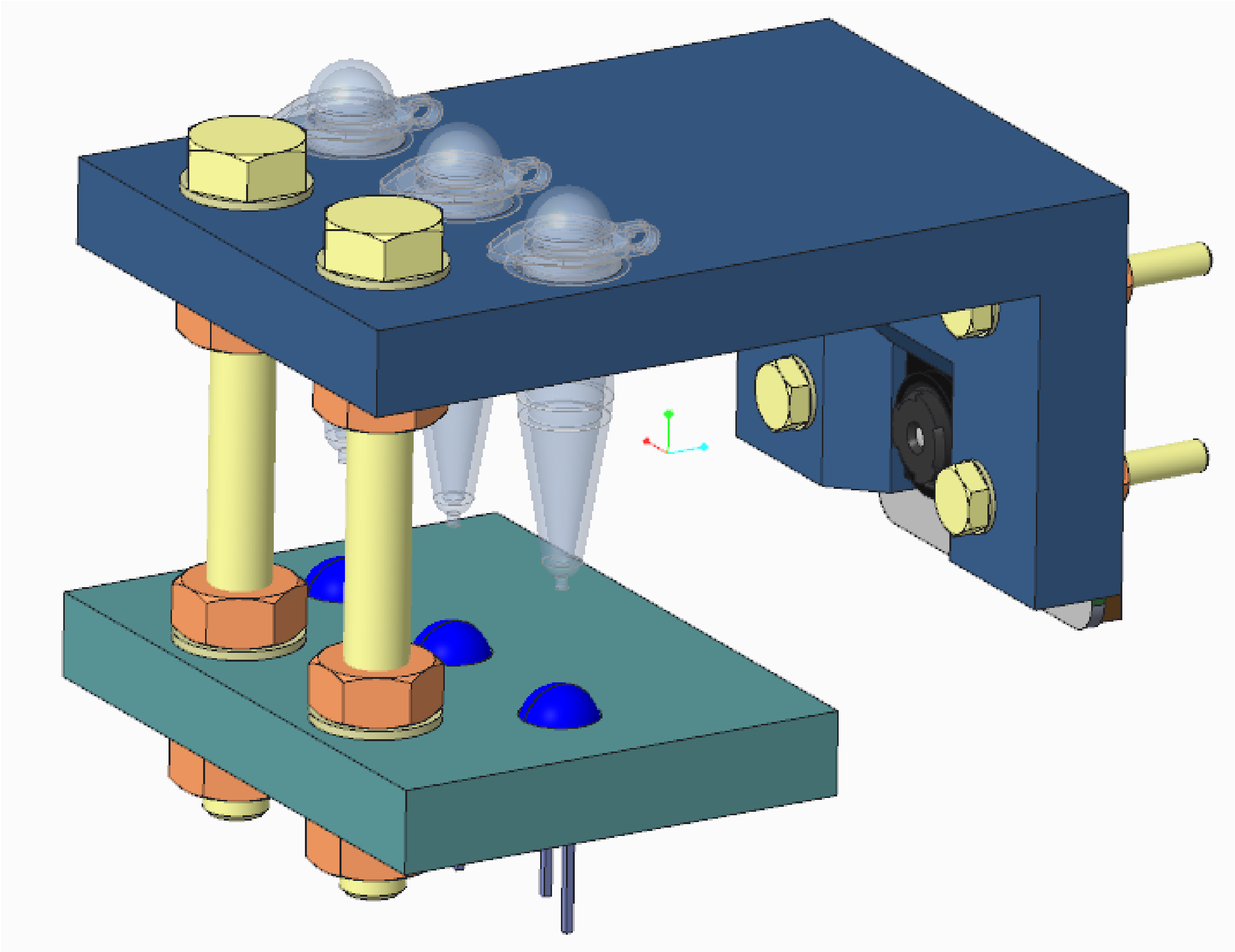

Supplementary Figure 33. CAD Mockup of Fluorescence Imaging Jig (Alternate view)

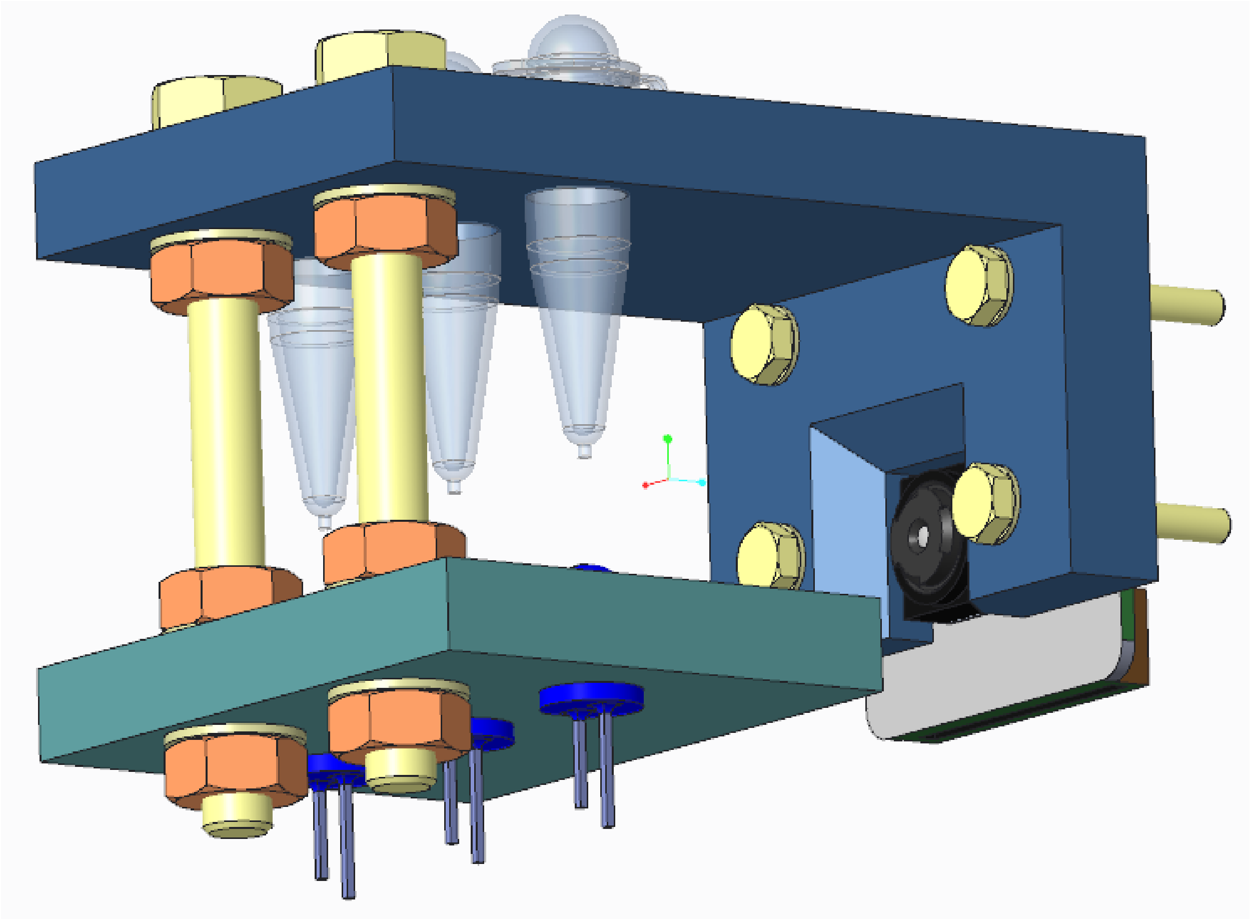

Supplementary Figure 34. Imaging jig with tubes.

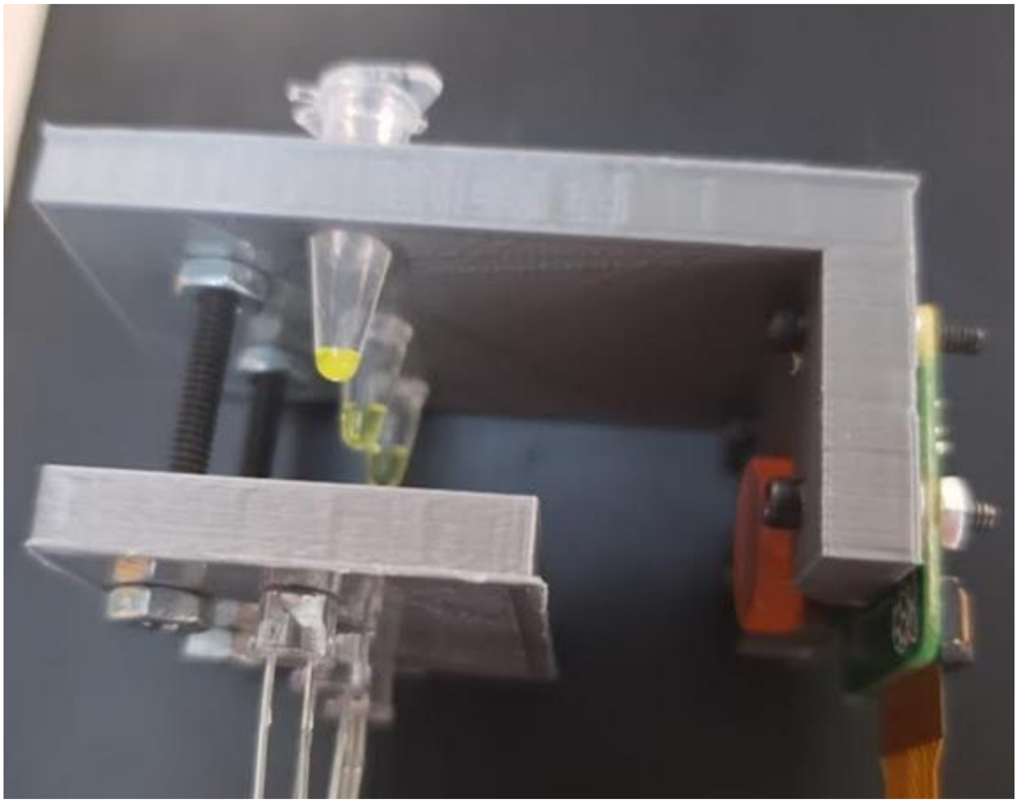

Supplementary Figure 35. Imaging jig (top view).

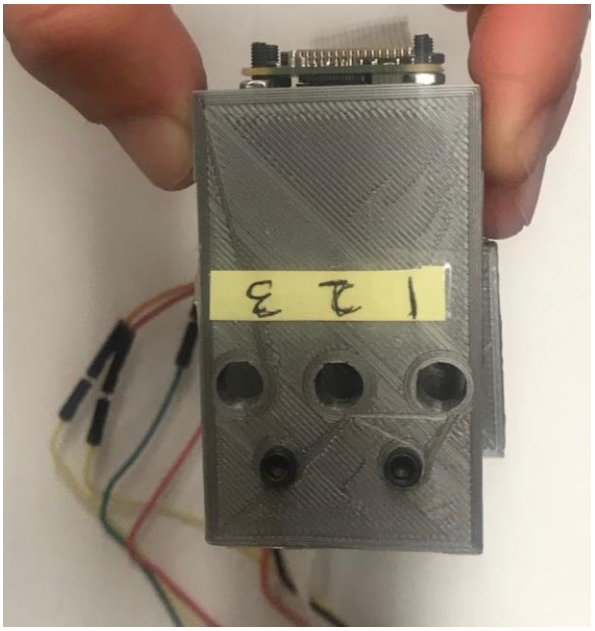

Supplementary Figure 36. Imaging Jig (side view)

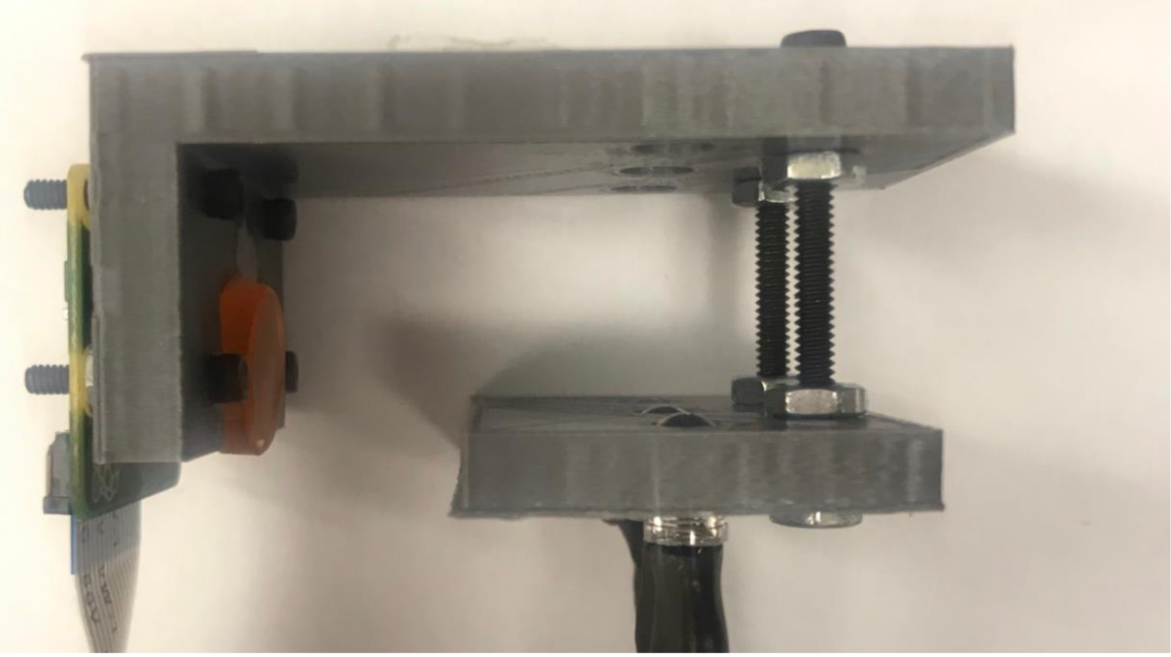

Supplementary Figure 37. Imaging Jig (front view).

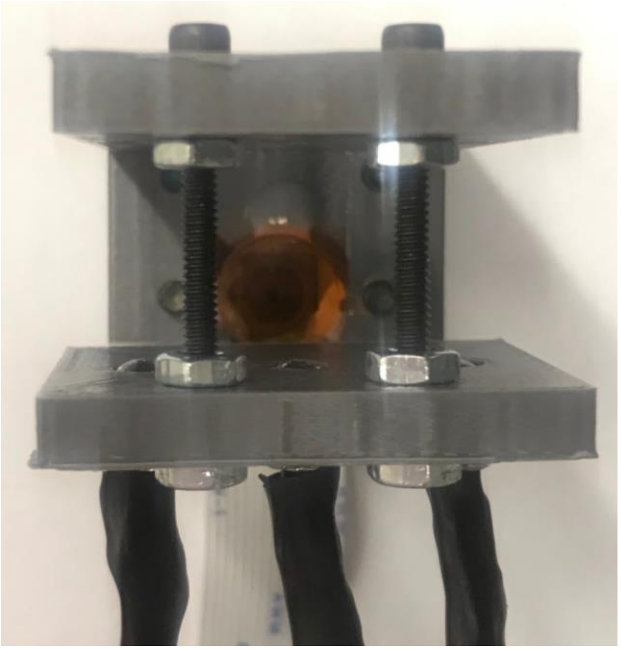

Supplementary Video 1. Fluorescence Imaging shows G4-Dark and Duplex-Dark switching structure under vacuum control with real-time plotting of fluorescence intensity. Available online.

Supplementary Video 2. Fluorescence Imaging shows G4-Dark and Duplex-Dark switching structure under vacuum control (With annotations and captions). Available online.

Supplementary Code 1. Python code for controlling Raspberry Pi for reaction monitoring and overlaying plots of fluorescence vs. time.

```
#!/usr/bin/env python3

# -*- coding: utf-8 -*-

"""

Created on Wed Jun 20 11:16:14 2018


@author: Lauren Aufdembrink

"""


####################################Code starts below here########################################

#@author: Lauren Aufdembrink


import time

from picamera import PiCamera

from gpiozero import LED

import numpy as np

from PIL import Image

import matplotlib.pyplot as plt

import os


Minutes=60*float(input('How long you would like to run in hours:'))

Intervals=float(input('How often do you want it to take photos in minutes:'))

PicturesTake=int(Minutes/Intervals)

Sample1=input('Name of sample in Slot 1:')

Sample2=input('Name of sample in Slot 2:')

Sample3=input('Name of sample in Slot 3:')

Data=input('Name file to save pixel intensities:')

ImageFolder=input('Name a folder to save tube images:')

PlotFolder=input('Name a folder to save plot images:')
```

```
OverlayFolder=input('Name a folder to save overlay images:')
pause=input('Press enter when you are ready to begin')
os.makedirs(ImageFolder)
os.makedirs(PlotFolder)
os.makedirs(OverlayFolder)


#######My function###########
def PixelAvg(image, Slot1, Slot2, Slot3):
#loading the image in:
    im=Image.open(image)
    #imarray=np.array(im)

#to take a subset from image for each tube
    #Slot1=imarray[50:240,1:427]
    #Slot2=imarray[52:240,427:854]
    #Slot3=imarray[50:240,855:1279]

#to go from an array back to an image:
#this is only necessary if you need to view your subset:
    #Slot1Im=Image.fromarray(Slot1)
    #Slot2Im=Image.fromarray(Slot2)
    #Slot3Im=Image.fromarray(Slot3)

#converting Images to grayscale:
    Slot1Gray=np.array(im.convert('L'),'f')[50:240,1:427]
    Slot2Gray=np.array(im.convert('L'),'f')[52:240,427:854]
    Slot3Gray=np.array(im.convert('L'),'f')[50:240,855:1279]
```

```
#finding the index of Max value of the image
#this will use that bright dot at the bottom of the tube to base my pixel subset off of
    Slot1MaxInd=np.unravel_index(np.argmax(Slot1Gray), (190,426))
    Slot2MaxInd=np.unravel_index(np.argmax(Slot2Gray), (188,427))
    Slot3MaxInd=np.unravel_index(np.argmax(Slot3Gray), (190,424))



#after finding the max value indexes above, taking the subset to average over:
    Slot1Pixel=Slot1Gray[(Slot1MaxInd[0]-30):(Slot1MaxInd[0]-10),(Slot1MaxInd[1]-
10):(Slot1MaxInd[1]+10)]
    Slot2Pixel=Slot2Gray[(Slot2MaxInd[0]-30):(Slot2MaxInd[0]-10),(Slot2MaxInd[1]-
10):(Slot2MaxInd[1]+10)]
    Slot3Pixel=Slot3Gray[(Slot3MaxInd[0]-30):(Slot3MaxInd[0]-10),(Slot3MaxInd[1]-
10):(Slot3MaxInd[1]+10)]

#now find the mean of your pixel selection:
    S1mean=np.mean(Slot1Pixel)
    S2mean=np.mean(Slot2Pixel)
    S3mean=np.mean(Slot3Pixel)

    Slot1.append(S1mean)
    Slot2.append(S2mean)
    Slot3.append(S3mean)

    return(S1mean, S2mean, S3mean, Slot1, Slot2, Slot3);

def ImageView(TubePic,PlotPic,i):
    background= Image.open(TubePic)
    overlay= Image.open(PlotPic)

    background.paste(overlay, (400,360))
```

```
    background.save(OverlayFolder+'/Ovly%s.jpg'%i, 'jpeg')

    return(background);

#insert the name of the file where the data is being stored once analyzed
DataFile=open(Data,'a+')

led= LED(12)
led2= LED(13)
led3= LED(19)
led.on()
led2.on()
led3.on()
camera=PiCamera()
camera.resolution=(1280,720)
camera.framerate=1
time.sleep(2)
camera.shutter_speed=750000
camera.exposure_mode='off'
camera.awb_mode='off'
camera.awb_gains=[3,1] #{red,blue]
camera.iso=100
time.sleep(2)
finalline='0 0 0'
Slot1=[]
Slot2=[]
Slot3=[]
Time=[]
led.off()
led2.off()
```

```
led3.off()
camera.stop_preview()
mydpi=100


for i in range(PicturesTake):
    time.sleep((Intervals*60)-3.5)
    Time.append(i*Intervals)
    led.on()
    led2.on()
    led3.on()
    camera.annotate_text=finalline
    camera.capture(ImageFolder+'/Rxn%s.jpg'%i)
    led.off()
    led2.off()
    led3.off()
    values=PixelAvg(ImageFolder+'/Rxn%s.jpg'%i, Slot1, Slot2, Slot3)
    S1=str(round(values[0]))
    S2=str(round(values[1]))
    S3=str(round(values[2]))
    DataFile.write(S1+'\t'+S2+'\t'+S3+'\n')
    finalline='S1:'+S1+'            S2:'+S2+'            S3:'+S3
    plt.plot(Time, Slot1,'g-', label=Sample1)
    plt.plot(Time, Slot2,'r--',label=Sample2)
    plt.plot(Time, Slot3, 'k^:', label=Sample3)
    plt.legend(loc=7, frameon=False)
    plt.xlim(0,Time[i]+5)
    plt.ylim(0,255)
    plt.xlabel('Time')
    plt.ylabel('Intensity (Au)')
```

```
    plt.title('Fluorescence Monitoring in Vacuum Chamber')
    plt.savefig(PlotFolder+'/Plot%s.jpg'%i, dpi=mydpi*0.75)
    plt.close()
    ImageView(ImageFolder+'/Rxn%s.jpg'%i,PlotFolder+'/Plot%s.jpg'%i, i)

led.off()
led2.off()
led3.off()
camera.stop_preview()
DataFile.close()
plt.plot(Time, Slot1,'co-', label=Sample1)
plt.plot(Time, Slot2,'ms--',label=Sample2)
plt.plot(Time, Slot3, 'g^:', label=Sample3)
plt.legend(loc=7, frameon=False)
plt.xlabel('Time')
plt.ylabel('Intensity (Au)')
plt.title('Fluorescence Monitoring in Vacuum Chamber')
plt.savefig('Fluorescence Monitoring in Vacuum Chamber')
plt.close()
```

Supplementary Code 2. Python code for reanalyzing collected images.

```
# -*- coding: utf-8 -*-
"""
Created on Tue Sep 24 18:19:38 2019

@author: aufde025
"""


######To reanalyze old images#######
import os
import numpy as np
from PIL import Image
import matplotlib.pyplot as plt

ImageFolder=input('Name of folder where Rxn Images are:')
path, dirs, files = next(os.walk(r"C:\Users\aufde025\Google Drive\EA-Lab_Sept2019\Nanomotor\7-26-19_dessication_run4_images"))
file_count = len(files)
Data=input('Name file to save pixel intensities:')
#insert the name of the file where the data is being stored once analyzed
DataFile=open(Data,'a+')

#######My function for image analysis############
def PixelAvg(image, Slot1, Slot2, Slot3):
#loading the image in:
    im=Image.open(image)
    #imarray=np.array(im)

#to take a subset from image for each tube
    #Slot1=imarray[50:240,1:427]
```

```
    #Slot2=imarray[52:240,427:854]
    #Slot3=imarray[50:240,855:1279]

#to go from an array back to an image:
#this is only necessary if you need to view your subset:
    #Slot1Im=Image.fromarray(Slot1)
    #Slot2Im=Image.fromarray(Slot2)
    #Slot3Im=Image.fromarray(Slot3)

#converting Images to grayscale:
    Slot1Gray=np.array(im.convert('L'),'f')[50:240,1:427]
    Slot2Gray=np.array(im.convert('L'),'f')[52:240,427:854]
    Slot3Gray=np.array(im.convert('L'),'f')[50:240,855:1279]


#finding the index of Max value of the image
#this will use that bright dot at the bottom of the tube to base my pixel subset off of
    Slot1MaxInd=np.unravel_index(np.argmax(Slot1Gray), (190,426))
    Slot2MaxInd=np.unravel_index(np.argmax(Slot2Gray), (188,427))
    Slot3MaxInd=np.unravel_index(np.argmax(Slot3Gray), (190,424))


#after finding the max value indexes above, taking the subset to average over:
    Slot1Pixel=Slot1Gray[(Slot1MaxInd[0]-30):(Slot1MaxInd[0]-10),(Slot1MaxInd[1]-
10):(Slot1MaxInd[1]+10)]
    Slot2Pixel=Slot2Gray[(Slot2MaxInd[0]-30):(Slot2MaxInd[0]-10),(Slot2MaxInd[1]-
10):(Slot2MaxInd[1]+10)]
    Slot3Pixel=Slot3Gray[(Slot3MaxInd[0]-30):(Slot3MaxInd[0]-10),(Slot3MaxInd[1]-
10):(Slot3MaxInd[1]+10)]

#now find the mean of your pixel selection:
```

```
    S1mean=np.mean(Slot1Pixel)
    S2mean=np.mean(Slot2Pixel)
    S3mean=np.mean(Slot3Pixel)

    Slot1.append(S1mean)
    Slot2.append(S2mean)
    Slot3.append(S3mean)

    return(S1mean, S2mean, S3mean, Slot1, Slot2, Slot3);
Slot1=[]
Slot2=[]
Slot3=[]
values=[]

for i in range(file_count):
    values=PixelAvg(ImageFolder+'/Rxn%s.jpg'%i, Slot1, Slot2, Slot3)
    DataFile.write(str(values[0])+'\t'+str(values[1])+'\t'+str(values[2])+'\n')
    values=[]

DataFile.close()
```

Supplementary Code 3. Python code for generating fluorescence plot overlays for existing image sets.

```
# -*- coding: utf-8 -*-
"""
Created on Tue Sep 24 10:14:15 2019

@author: aufde025
"""


import csv
import matplotlib.pyplot as plt
import os
from PIL import Image





#####Code to make the new graphs##########
Sample1=input('Name of sample in Slot 1:')
Sample2=input('Name of sample in Slot 2:')
Sample3=input('Name of sample in Slot 3:')
PlotFolder=input('Name a folder to save plot images:')
ImageFolder=input('Name a folder where rxn images are:')
OverlayFolder=input('Name a folder to save new overlay images:')
Title=input('What do you want your graph title:')
Interval=float(input('How often did your script take photos (in mins):'))
pause=input('Press enter when you are ready to begin')
os.makedirs(PlotFolder)

#####function to make overlays#######
def ImageView(TubePic,PlotPic,i):
```

```
    background= Image.open(TubePic)
    overlay= Image.open(PlotPic)

    #added dpi to plt saving so I no longer need to resize
    #wsize=int(min(background.size[0], background.size[1])*0.75)
    #wpercent=(wsize/float(overlay.size[0]))
    #hsize=int((float(overlay.size[1])*float(wpercent)))

    #simage=overlay.resize((wsize, hsize))
    #mbox=background.getbbox()
    #sbox=simage.getbbox()

    #box=(mbox[2]-sbox[2],mbox[3]-sbox[3])
    background.paste(overlay, (400,360))
    background.save(OverlayFolder+'/Ovly%s.jpg'%i, 'jpeg')
    #background.show(background.thumbnail((700,600))) #this resizes it so it fits on Raspi screen

    return(background);

#######code to make new graph#########
Time=[]
i=0
mydpi=100
val1=[]
val2=[]
val3=[]
with open('7-26-19_dessication_run4_intensities') as csvfile:
    readCSV=csv.reader(csvfile,delimiter='\t')
    for row in readCSV:
        Time.append(i*Interval)
```

```
val1.append(float(row[0]))
val2.append(float(row[1]))
val3.append(float(row[2]))
plt.plot(Time, val1,'g-', label=Sample1)
plt.plot(Time, val2,'r--',label=Sample2)
plt.plot(Time, val3, 'k^:', label=Sample3)
plt.legend(loc=7, frameon=False)
plt.xlim(0,Time[i]+5)
plt.ylim(0,50)
plt.xlabel('Time')
plt.ylabel('Intensity (Au)')
plt.title(Title)
plt.savefig(PlotFolder+'/Plot%s.jpg'%i, dpi=mydpi*0.75)
i=i+1
ImageView(ImageFolder+'/Rxn%s.jpg'%i,PlotFolder+'/Plot%s.jpg'%i, i)
plt.close()
```

Supplementary Table 1. Melting temperature of the fluorescent nucleic acid systems in low (0.8 M) and high (8 M) concentrations of sodium perchlorate with varying concentrations of oligonucleotide. **G4-Dark** is presented in both duplex and G4 sections; at low salt, the duplex-G4 transition is observed, and at high salt, the G4-single stranded transition is observed.

	Duplex T_M (°C)			G4 T_M (°C)		
	**Duplex-Dark**		**G4-Dark**		**Fluorescein-G4-Quencher**	
Oligo Concentration	0.8 M $NaClO_4$	8 M $NaClO_4$	0.8 M $NaClO_4$	8 M $NaClO_4$	0.8 M $NaClO_4$	8 M $NaClO_4$
0.1 µM	74.5 ±1.0	Low	67.5 ±1.3	73.9 ±1.5	82.9 ±1.1	72.8 ±1.1
1 µM	78.1 ±1.0	Low	70.4 ±0.8	72.3 ±1.0	82.7 ±1.1	72.4 ±1.2
10 µM	81.5 ±1.0	Low	75.1 ±0.8	71.7 ±0.9	83.1 ±1.4	71.7 ±0.9

Supplementary Table 2. Thermal midpoints of **G4-SwitchR.** Measurements were obtained by UV-vis monitoring of A-$_{260}$ and A-$_{295}$.

	T_M (°C)	
[$NaClO_4$] (M)	duplex	G4
0.1	70.5	High
1	73.6	High
2	66.0	75.4
3	56.9	69.7
4	47.9	66.7
5	35.5	60.8
6	29.8	58.8
7	Low	53.4
8	Low	52.5
Saturated (ca. 9 M)	Low	51.8

Supplementary Figure 38. Comparative kinetic measurements of oxidation of Amplex Red by **G4Redox** and **G4-SwitchR** in the presence of varying concentrations of $NaClO_4$ at different time points. A) 0 minutes. B) 10 minutes. C) 20 minutes. D) 30 minutes.

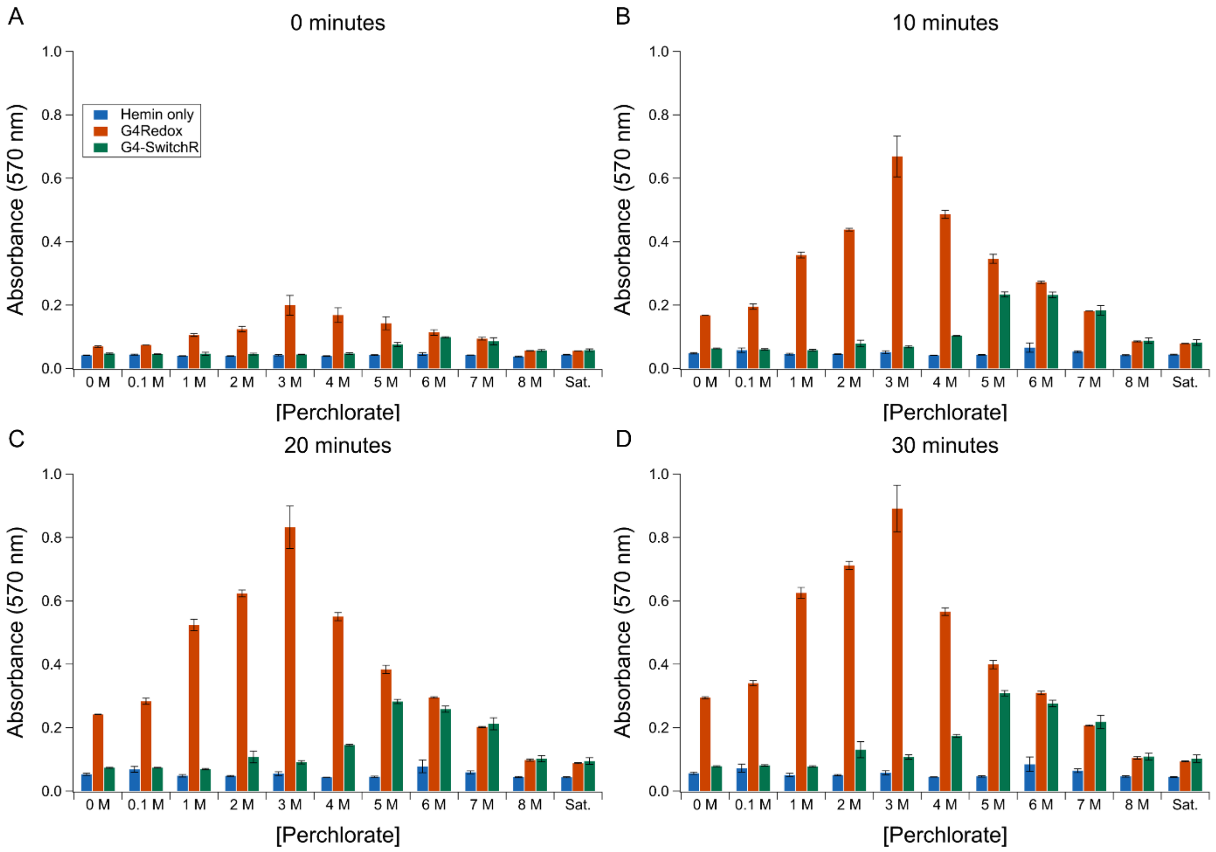

Supplementary Figure 39. UV-visible spectra of hemin interacting with **G4Redox** and **G4SwitchR** in varying sodium perchlorate concentrations. (a) In 0.1 M $NaClO_4$ **G4SwitchR** is in the duplex state and does not interact with hemin. (b) In 5 M $NaClO_4$ **G4-SwitchR** changes to the G quadruplex conformation (**G4Redox**) and interacts with hemin, and a Soret peak is observed. (c) In 8 M $NaClO_4$ **G4-SwitchR** is still in the G quadruplex state, but it has a weaker interaction with hemin. Hemin and oligonucleotide concentrations were the same as those used in the Amplex Red oxidation assay.

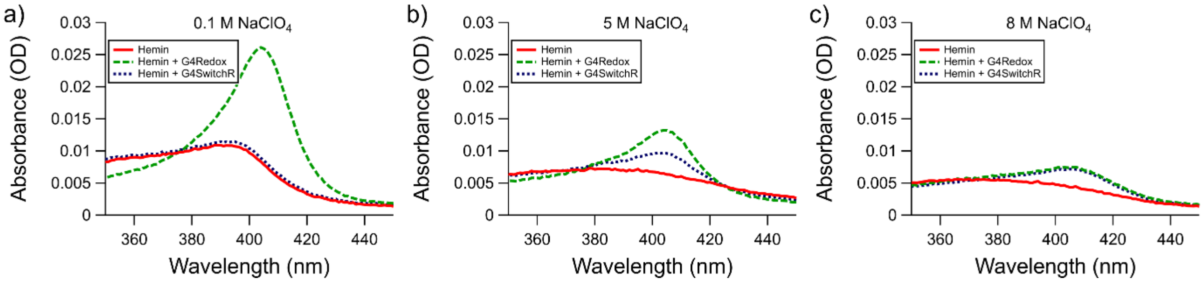

Supplementary Figure 40. CD spectra of **G4Redox** and **G4-SwitchR** in the presence and absence of hemin. (a) In 0.1 M $NaClO_4$ **G4Redox** is stabilized into a parallel G quadruplex with the addition of hemin as indicated by the strong peak at 265nm. **G4-SwitchR** does not interact with hemin and maintains a peak at 260 nm. (b) In 5 M $NaClO_4$ **G4-SwitchR** switches conformation from duplex to G quadruplex and interacts with hemin producing a peak at 265 nm. (c) In 8 M $NaClO_4$ **G4Redox** exists in an antiparallel conformation (peak at 295nm) which may account for the reduced peroxidase activity seen in 8 M to saturated (~9.5 M) $NaClO_4$. **G4Redox-Comp** was subtracted from all **G4-SwitchR** spectra.

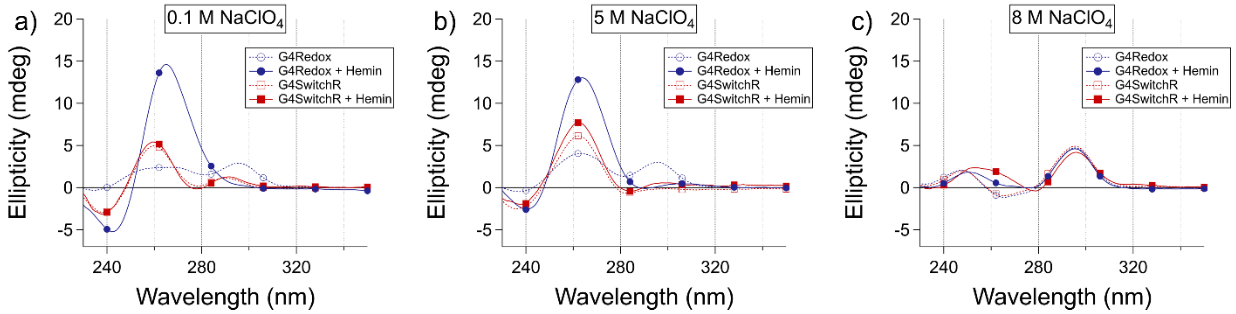

Supplementary Figure 41. UV-vis spectra of **G4Redox** and hemin in varying sodium perchlorate concentrations. Spectra are shown for (a) 0.1 M, (b) 5 M, and (c) 8 M sodium perchlorate. (d-f) enlarged portion of the Soret peak from spectra shown in panels (a-c). The concentration of DNA is not appreciably different following extended (1H) incubation or centrifugation after incubation. Consistent with the propensity of porphyrins to aggregate, partial removal of hemin from solution occurs following extended (10 minute) centrifugation, but this is observed in both low- and high-salt solution, and the G-quadruplex-dependent Soret absorbance (**Supplementary Figure 39**) shows hemin is bound to DNA.

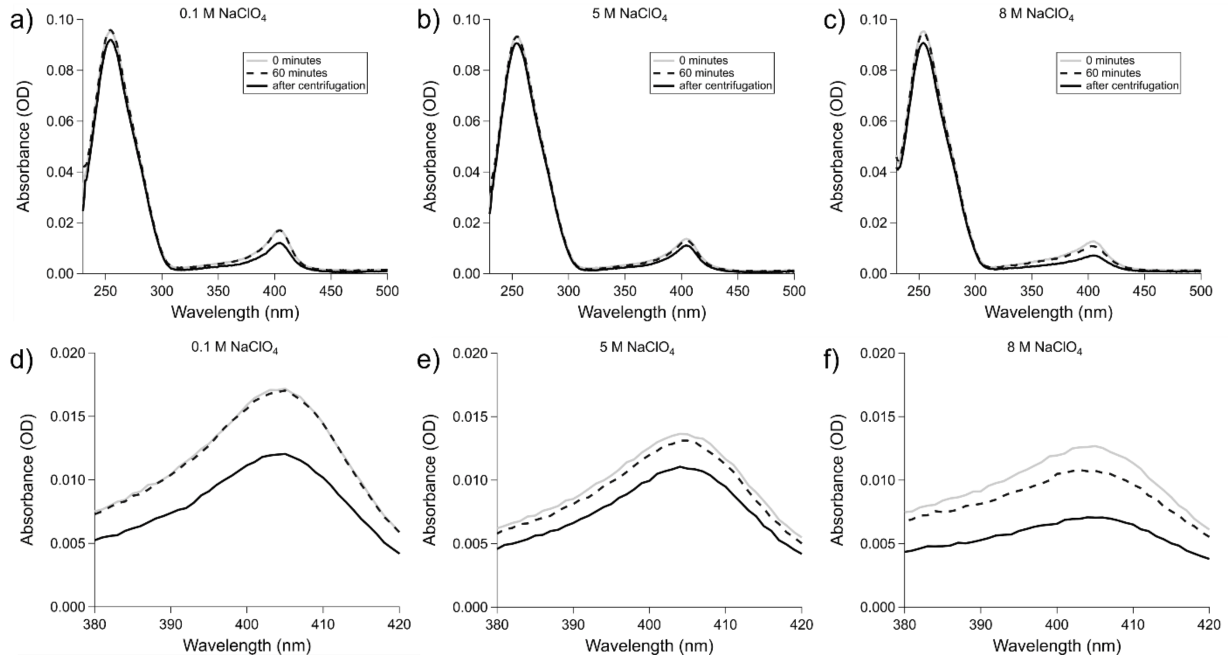

Supplementary Figure 42. **G4-SwitchR**-hemin complexes catalyze oxidation of TMB and ABTS. Oxidation of (a) TMB and (b) ABTS by hemin alone or in solution with **G4Redox** or **G4-SwitchR**.

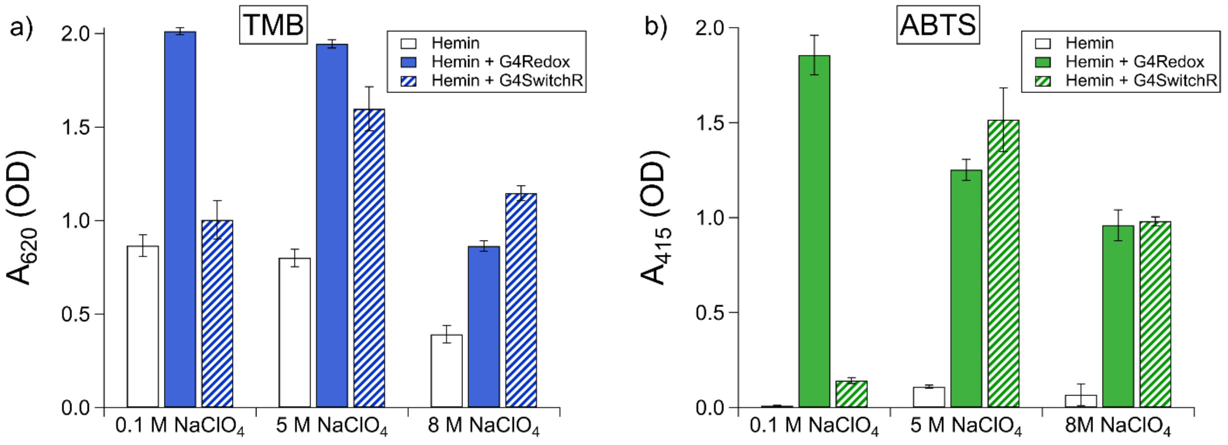

Supplementary Figure 43. A_{295}-monitored melting curve of the **G4-SwitchR** system. Melt samples consisted of 5 µM **G4Redox** and 5 µM **G4Redox-Comp** in 5 mM sodium phosphate buffer, pH 7.4. A_{295} increases with G4 formation. The trace initially starts at low values when **G4Redox** is duplexed with **G4Redox-Comp**, increases between ca. 20 and 40 °C as the duplex separates and **G4Redox** forms a G-quadruplex, then decreases between ca. 40 and 70 °C as the **G4Redox** G-quadruplex unfolds.

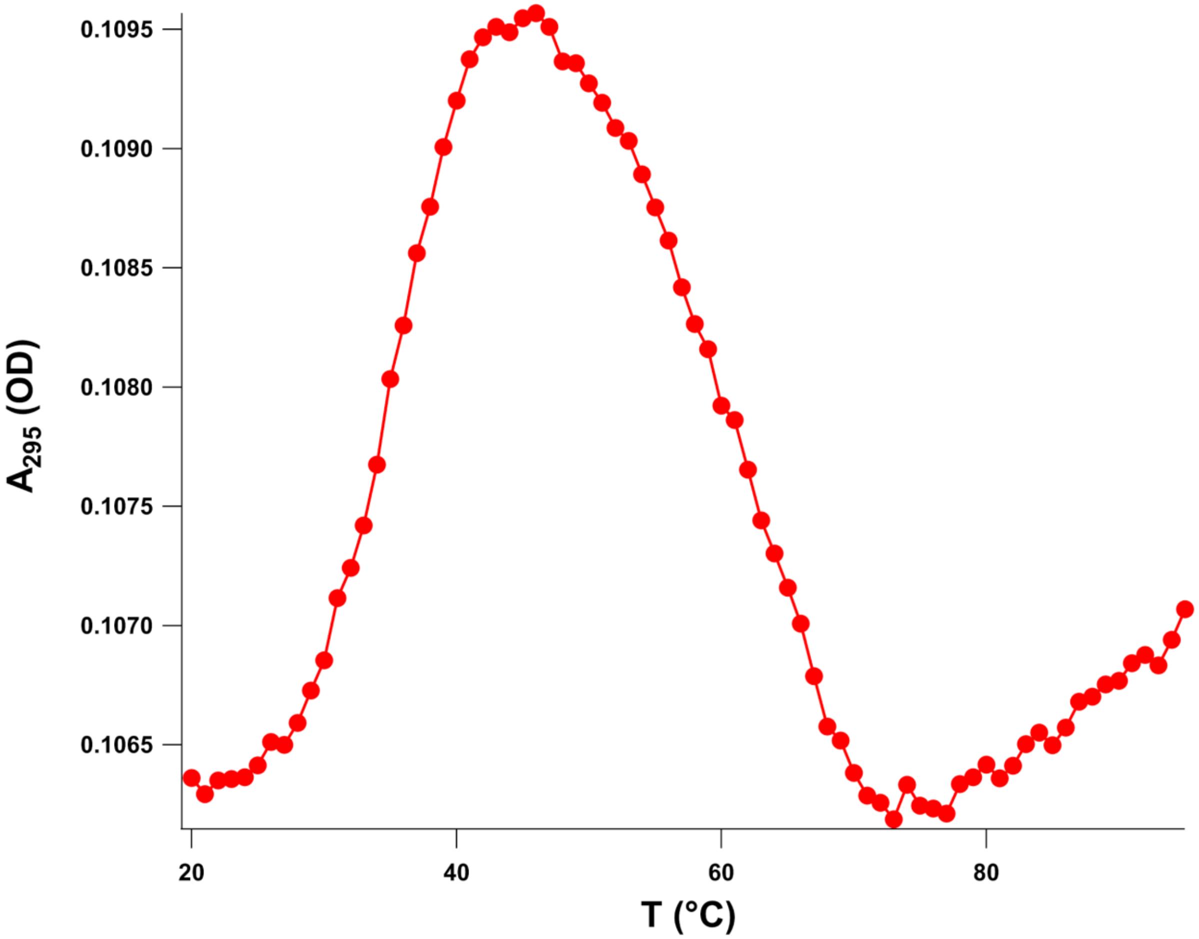

Supplementary Figure 44. A_{260}-monitored melting curve of the **G4-SwitchR** system. A_{260}, which increases with unstacking of bases, exhibits the same transitions as the A_{295} trace. A_{260} initially starts at low values when **G4Redox** is duplexed with **G4Redox-Comp**, increases between ca. 20 and 40 °C as the duplex separates and **G4Redox** forms a G-quadruplex. A_{260} then increases further between ca. 40 and 70 °C as the **G4Redox** G-quadruplex unfolds. Melt samples consisted of 5 µM **G4Redox** and 5 µM **G4Redox-Comp** in 5 mM sodium phosphate buffer, pH 7.4.

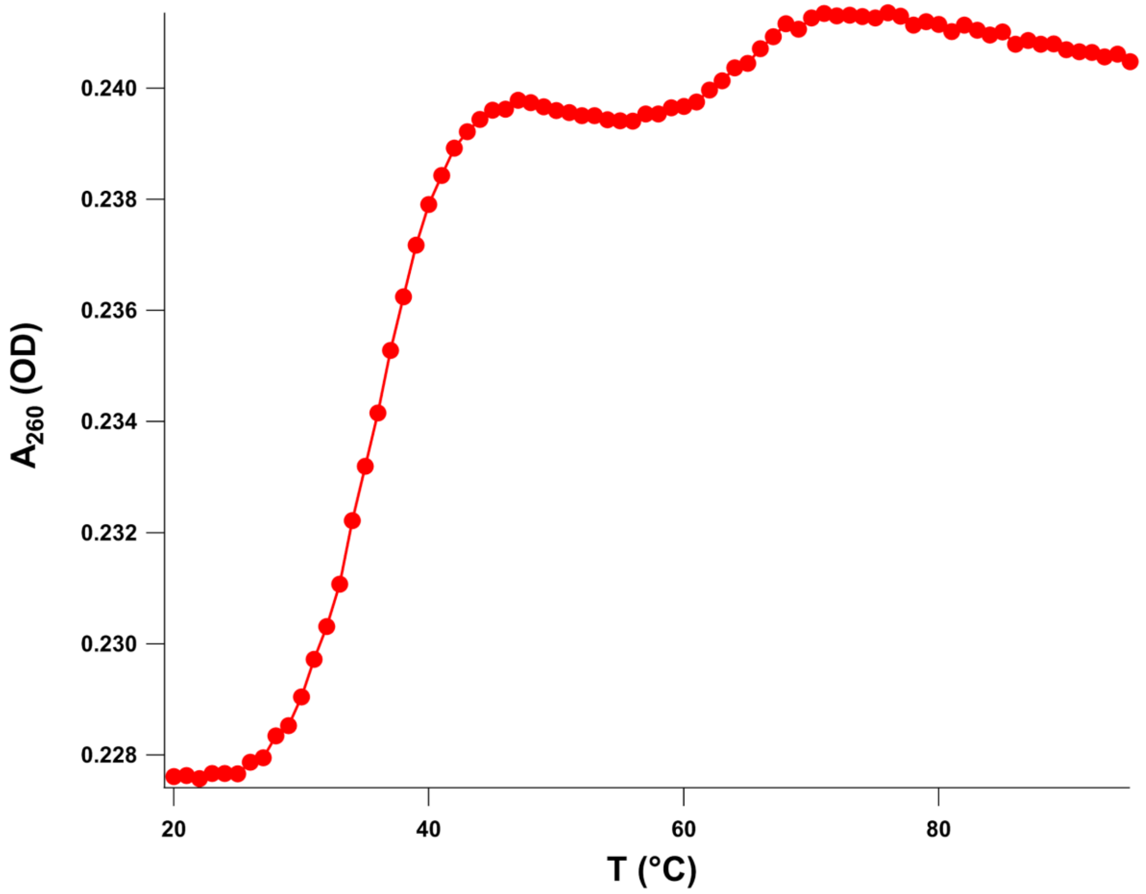

Supplement: Supplementary file 1 — Supporting Information [file CBIC-23-0-s002.pdf]
